# Cathepsin L regulates oocyte meiosis and preimplantation embryo development

**DOI:** 10.1111/cpr.13526

**Published:** 2023-07-07

**Authors:** Mohamed Aboul Ezz, Masashi Takahashi, Rocío Melissa Rivera, Ahmed Zaky Balboula

**Affiliations:** ^1^ Department of Theriogenology, Faculty of Veterinary Medicine Mansoura University Mansoura Egypt; ^2^ Division of Animal Sciences University of Missouri Columbia Missouri USA; ^3^ Research Faculty of Agriculture Hokkaido University Hokkaido Japan

## Abstract

Early embryonic loss, caused by reduced embryo developmental competence, is the major cause of subfertility in humans and animals. This embryo developmental competence is determined during oocyte maturation and the first embryo divisions. Therefore, it is essential to identify the underlying molecules regulating these critical developmental stages. Cathepsin L (CTSL), a lysosomal cysteine protease, is involved in regulating cell cycle progression, proliferation and invasion of different cell types. However, CTSL role in mammalian embryo development is unknown. Using bovine in vitro maturation and culture systems, we show that CTSL is a key regulator for embryo developmental competence. We employed a specific CTSL detection assay in live cells to show that CTSL activity correlates with meiotic progression and early embryo development. Inhibiting CTSL activity during oocyte maturation or early embryo development significantly impaired oocyte and embryo developmental competence as evidenced by lower cleavage, blastocyst and hatched blastocyst rates. Moreover, enhancing CTSL activity, using recombinant CTSL (rCTSL), during oocyte maturation or early embryo development significantly improved oocyte and embryo developmental competence. Importantly, rCTSL supplementation during oocyte maturation and early embryo development significantly improved the developmental competence of heat‐shocked oocytes/embryos which are notoriously known for reduced quality. Altogether, these results provide novel evidence that CTSL plays a pivotal role in regulating oocyte meiosis and early embryonic development.

## Graphical abstract

Schematic model for cathepsin L role in regulating oocyte meiosis and early embryo development.

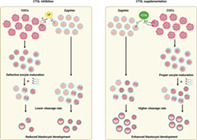

## 1 INTRODUCTION

Embryo mortality is a major cause of subfertility in humans^1^ and animals.^2^ In most mammalian species, including humans, embryo losses are estimated to be 20%–40% with at least two third of these embryos are lost during the early preimplantation period of pregnancy.^3^, ^4^ Successful implantation requires a viable embryo (at the blastocyst developmental stage), a receptive endometrium, as well as an embryo–endometrium effective crosstalk.^5^ To evolve a compartmentalized mammalian blastocyst ready for implantation, a highly organized sequence of fundamental events must occur. These events include fertilization, oocyte meiosis completion, a series of cleavage divisions, embryonic genome activation, compaction, cavitation (blastocoel formation) and cellular differentiation into the trophectoderm and inner cell mass.^6^, ^7^ The quality of the blastocyst, is largely dependent on the quality of the oocyte^8^, ^9^, ^10^, ^11^ and positively correlated with implantation success and live births.^12^, ^13^, ^14^ A better understanding of the molecular mechanisms that govern these events is crucial in mitigating early pregnancy losses in humans and animals.^15^

Cathepsins are well known lysosomal proteases.^16^ According to the amino acid present at their active site, cathepsins are categorized into three subfamilies: aspartic (cathepsin A and G), cysteine (cathepsin B, C, F, H, K, L, O, S, V, W and X/Z) and serine (cathepsin D and E) proteases.^17^, ^18^ These proteases orchestrate a variety of cell functions including immune response, autophagy, growth, as well as development.^19^ Of relevance, we have previously demonstrated that the intracellular activities of cathepsin (CTS)B (CTSB) and CTSK are inversely correlated with the quality and development of oocytes and preimplantation embryos.^20^, ^21^, ^22^, ^23^, ^24^, ^25^, ^26^, ^27^, ^28^ Cathepsin L (CTSL) has been reported to be involved in the proliferation and differentiation of embryonic stem cells^29^ as well as implantation and placental formation processes in several species.^30^, ^31^, ^32^, ^33^ However, little is known about the function of this cysteine protease in mammalian oocyte meiosis and embryo development. Using a bovine model, we showed that CTSL activity is correlated with oocyte and embryo quality and regulating such activity is an efficient approach to improve the quality and developmental competence of oocytes and preimplantation embryos. Importantly, CTSL upregulation significantly rescued, at least in part, the developmental competence of heat‐shocked oocytes/embryos (notoriously known for reduced quality).

## 2 MATERIALS AND METHODS

### 2.1 Chemicals

All chemicals were purchased from Millipore Sigma (St. Louis, MO, USA) unless otherwise stated.

### 2.2 In vitro maturation, fertilization and culture

Bovine embryos were produced in vitro as we previously described.^34^ Briefly, bovine ovaries were obtained from a local abattoir and transported to the laboratory within 2–3 h in a 0.9% (w/v) warm sodium chloride (NaCl) solution. The ovaries were slashed, and cumulus‐oocyte complexes (COCs) were collected into oocyte collection medium (M‐199 with Hank's salts and supplemented with 2% [v/v] calf bovine serum [ATCC, #30–2030], 1 mM L‐glutamine, 0.2 USP/mL heparin, 100 IU/mL penicillin and 0.1 mg/mL streptomycin). Freshly collected immature COCs were microscopically categorized into nine morphological groups before being pooled into either good or poor quality groups as previously described.^35^ Briefly, the COCs were categorized into nine morphological groups: Group I, II and III (i.e., fully grown oocytes surrounded by more than three compact cumulus layers with homogenous medium brown cytoplasm) were pooled together as good quality COCs, whereas groups IV–IX (i.e., fully grown oocytes showing either abnormal or no cumulus cells and/or inhomogeneous darker or paler cytoplasm) were pooled together as poor quality COCs. Unless otherwise specified, only good quality COCs were used. In vitro maturation (IVM) was performed by culturing the COCs (in groups of ~50) in 500 μL/well oocyte maturation medium (OMM) (M‐199 with Earle's salts supplemented with 10% [v/v] bovine calf serum, 1 mM L‐glutamine, 0.2 mM sodium pyruvate, 20 μg/mL follicle stimulating hormone [FSH; Folltropin‐V®, Vetoquinol, TX, USA], 2 μg/mL β‐estradiol and 10 μg/mL gentamycin) in Nunclon™ four well plates (Fisher Scientific, Pittsburgh, PA) at either 38.5°C (the homoeothermic temperature of cows; control group) or 41°C (heat shock [HS] group) in a humidified atmosphere containing 5% CO_2_.^36^

After 22–24 h of IVM, the COCs were washed in HEPES‐TALP medium (HEPES‐TL [Caisson Laboratories Inc., Smithfield, Utah, USA] supplemented with 3 mg/mL bovine serum albumin [BSA] fraction V, 0.2 mM sodium pyruvate and 7.5 μg/mL gentamycin), then transferred (in groups of ~30) to 500 μL/well IVF‐TALP medium (IVF‐TL [Caisson] supplemented with 6 mg/mL essential fatty acid free‐BSA [EFAF‐BSA], 0.2 mM sodium pyruvate, 10 μg/mL heparin and 7.5 μg/mL gentamycin) in four‐well plates. For in vitro fertilization (IVF), frozen semen straws from proven fertility Angus bull were thawed at 38.5°C and purified by density centrifugation using Isolate (Irvine Scientific, Santa Ana, CA) at 1000 × *g* for 5 min, then washed in HEPES‐TALP medium at 195 × *g* for 5 min. The sperm pellet was resuspended in IVF‐TALP medium, then added to the matured COCs at approximately 1 × 10^6^ spermatozoa per well, and co‐cultured at 38.5°C in a humidified atmosphere containing 5% CO_2_.

Twelve hours post‐insemination (hpi), putative zygotes were stripped of their cumulus cells by vortexing for 3.5 min, washed in HEPES‐TALP medium, then transferred (in groups of ~30) to 500 μL/well modified potassium simplex optimized medium (KSOM) supplemented with 3 mg/mL EFAF‐BSA, essential and non‐essential amino acids and 2.5 μg/mL gentamycin in four‐well plates. In vitro culture (IVC) proceeded for 7–8 days at 38.5°C in a humidified atmosphere containing 5% CO_2_, 5% O_2_ and 90% N_2_. HS embryos were only exposed to 41.0°C for the first 12 h of IVC period (i.e., from 12 to 24 hpi), then the temperature was kept at 38.5°C for the remainder of IVC duration.^37^ The cleavage, blastocyst (i.e., the sum of early blastocyst [EBL], blastocyst [BL], expanded blastocyst [XBL] and hatched blastocyst [HBL]), and HBL rates were assessed at 60, 180 and 204 hpi, respectively.

Lactoferrin from bovine milk (LF; #L9507, Sigma) was dissolved in water and added to IVM/IVC medium at 10, 100 or 1000 μg/mL to inhibit CTSL activity in good quality oocytes/embryos while recombinant human active CTSL (rCTSL; #ab198444, Abcam, MA, USA) was supplemented to IVM/IVC medium at 100, 200 or 400 ng/mL to enhance CTSL activity in good quality oocytes/embryos. The cellular uptake of the recombinant cathepsins has been previously demonstrated.^38^, ^39^

### 2.3 Detection of intracellular CTS activity

The intracellular activity of CTSL was assayed in COCs, denuded oocytes or embryos using Magic Red® CTSL detection kit (#941, Immunochemistry Technologies, LLC, Bloomington, MN, USA) according to the manufacturer's protocols. Briefly, the targeted cells (COCs, denuded oocytes or embryos) were incubated in the reaction mix (2 μL of DMSO diluted stock solution in 500 μL culture medium [either OMM or KSOM]) for 20–25 min at 38.5°C in a humidified atmosphere containing 5% CO_2_. For DNA labelling, 1 μg/mL Hoechst 33342 (#639, Immunochemistry Technologies) was added to the cell suspension and co‐incubated for further 5–10 min. The stained cells were rinsed twice in phosphate buffered saline (PBS, 10 mM potassium phosphate [KPO_4_], 0.9% [w/v] NaCl, pH 7.4) to which 1 mg/mL polyvinylpyrrolidone (PVP) was added (PBS‐PVP). The COCs were then mounted onto a glass slide using a Vectashield® mounting medium (Vector Laboratories Inc., CA, USA), while oocytes/embryos were transferred to a pre‐heated (at 38.5°C) 50 μL drop of PBS‐PVP under oil in a 35 mm glass‐bottom dish (MatTek Corporation, Ashland, MO, USA). Subsequently, the cells were imaged at 200X using a DMi8 S Platform inverted epifluorescence microscope supported with LAS X software (Leica Microsystems Inc, Buffalo Grove, IL); Magic Red (CTSL activity) was observed as red colour using an excitation filter of 550–590 nm, while Hoechst 33342 (DNA) was observed as blue colour using an excitation filter of 365 nm. The images were captured, then fluorescence emission (red = 592–668 nm; blue = 435–485 nm) intensity (pixels) was analysed using Image J software (FIJI®; National Institutes of Health, Bethesda, MD, USA); the mean values of CTSL fluorescent intensity (i.e., sum of pixel values/area of selection) were applied for measuring CTSL activity in the defined cells. CTSL fluorescent intensity was measured in the whole area of oocytes/embryos including their zona pellucida. Due to the unequal cumulus layers surrounding COCs, the measurement was applied to the area of oocyte (including zona pellucida) as well as the first two cumulus cell layers. When comparing CTSL activity levels between embryonic stages, we used raw integrated density values (i.e., sum of pixel values within the selection) to account for blastocoel presence in blastocyst stage embryos. Of note, the images of CTSL activity within the same experiments were captured under the same image setting. The oocytes were denuded from their cumulus cells using 1% hyaluronidase solution with vortexing for 3.5 min.^25^ In a similar way to CTSL detection assay, the intracellular activity of CTSB and CTSK was detected in bovine COCs using Magic Red® CTSB detection kit (#938, Immunochemistry Technologies) and CTSK detection kits (#940, Immunochemistry Technologies).

### 2.4 Cell livability assessment

The percentage of dead cells in COCs/embryos was determined on the basis of plasma membrane integrity using a supravital staining (propidium iodide [PI]; #P4170, Sigma) as previously described^40^ with minor modifications. Briefly, COCs/embryos were incubated in a culture medium (OMM/KSOM), supplemented with 10 μg/mL PI as well as 1 μg/mL Hoechst 33342, for 10–15 min at 38.5°C in a humidified atmosphere containing 5% CO_2_. The samples were then washed twice in PBS‐PVP, mounted onto a glass slide, and observed using epifluorescence microscopy. Using an excitation filter of 550–590 nm, the dead cells (i.e., with degenerated plasma membrane) showed a red fluorescence (PI‐positive), while the viable cells (i.e., with intact plasma membrane) did not show a red fluorescence (PI‐negative); Hoechst 33342 was incorporated in such reaction for DNA labelling (blue). To confirm that the red colour appeared in PI‐positive cells was not a result of autofluorescence phenomenon, positive and negative controls were implicated in each experimental design. Positive control COCs/embryos were incubated in RQ1 RNase‐free DNase (50 U/mL) at 38.5°C in a humidified atmosphere containing 5% CO_2_ for 1 h prior to running PI/Hoechst 33342 staining reaction. On the other hand, negative control COCs/embryos were incubated with Hoechst 33342 only (no PI staining was added to such reaction).

### 2.5 Blastocyst total cell number assessment

Blastocysts were harvested at 180 hpi and washed twice in PBS‐PVP. The nuclei of blastocysts were then stained via Hoechst 33342, mounted onto a glass slide, and observed under epifluorescence microscope with an excitation filter of 365 nm. Z‐stacks were reconstructed into a 3D structure for every blastocyst and the total cell number (i.e., the number of nuclei) was determined using a Microscopy Image Analysis Software (Imaris® 9.9 Release, Bitplane AG, Zurich, Switzerland).

### 2.6 Statistical analysis

Each experiment was repeated 3–4 independent times on different occasions. The data were collected, organized, summarized and presented as mean ± standard error of the mean (SEM). D'Agostino–Pearson omnibus normality test was performed to confirm the normal distribution of the numerical data before statistical analysis. The percentage data were transformed by arcsine formula before conducting analysis. The statistical analysis was performed using SAS software version 9.4 (SAS Institute, Cary, NC). The number of replicates was included in our statistical model. GraphPad Prism version 9.3.1 (GraphPad Software®, San Diego, CA, USA) was employed for figures' designing. The statistical tests used for data analysis of each experiment are specified below each graph. The results were considered statistically significant at *p* < 0.05.

## 3 RESULTS

### 3.1 The intracellular CTSL activity correlates with COCs and oocytes quality

**FIGURE 1:**
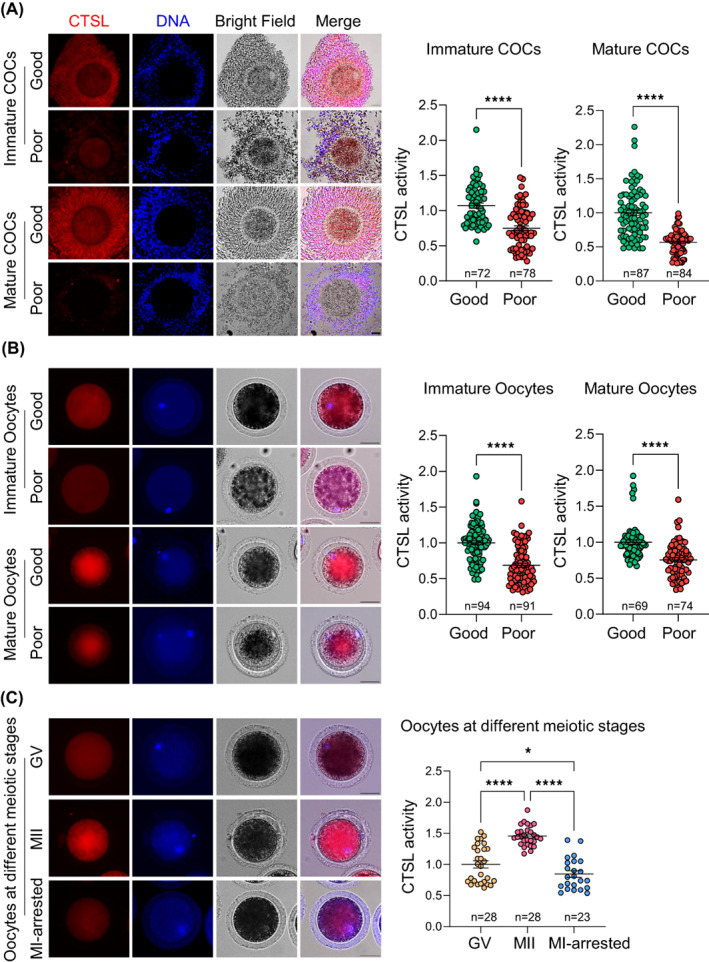
Intracellular CTSL activity relates to the quality of COCs/oocytes. Detection of intracellular CTSL activity in good and poor quality bovine COCs (A) or denuded oocytes (B) either pre‐ or post‐IVM conduction. (C) Quantification of CTSL activity during the different meiotic stages of bovine oocytes. CTSL activity represents the mean values of fluorescence intensity in the indicated cells. Scale bar represents 50 μm. The data of four independent experiments are presented as mean ± SEM. The total number of analysed COCs/oocytes is specified in each graph. Asterisks denote a significant variance (* means *p* < 0.05, and **** means *p* < 0.0001) between the different groups when compared using either Student's *t*‐test (A & B) or one‐way ANOVA followed by Tukey's multiple comparisons test (C). The number of replicates was included in our statistical model; there was a significant variance (*p* = 0.0089) between the different replicates (C). COCs, cumulus‐oocyte complexes; CTSL, cathepsin L; GV, germinal vesicle; IVM, in vitro maturation; MII, metaphase II; SEM, standard error of the mean.

To investigate the association between the quality of COCs/oocytes and intracellular CTSL activity, we first compared the levels of active CTSL in both good and poor quality immature COCs and identified lower CTSL activity in the poor quality immature COCs (*p* < 0.0001; Figure 1A). To clarify whether the reduction in CTSL activity could be corrected through the incorporation of these COCs into the physiological maturation process, both immature good and poor quality COCs were subjected to the routine IVM procedures followed by CTSL activity detection. In a similar pattern to the immature COCs, the poor quality mature COCs had lower intracellular CTSL activities when compared to their good quality counterparts (*p* < 0.0001; Figure 1A). To elucidate whether the disturbances in CTSL activities in relation to COCs quality is attributed to the oocytes and/or the cumulus cells, oocytes were denuded of their granulosa cells, either pre‐ or post‐IVM, and CTSL activity was assayed. In line with what we observed in COCs, CTSL activity was lower in both immature and mature denuded poor quality oocytes when compared to the good quality oocytes (*p* < 0.0001; Figure 1B). We then investigated whether CTSL activity is altered in relation to oocyte meiotic progression by measuring CTSL activity at various stages of oocyte meiosis. Results showed that metaphase II (MII) oocytes (i.e., the oocytes showing two sets of DNA with chromosomal alignment at MII plate^41^) had higher CTSL activities than prophase I‐arrested germinal vesicle (GV) oocytes or those failed to reach MII and were arrested at MI stage (*p* < 0.0001; Figure 1C).

**FIGURE 2:**
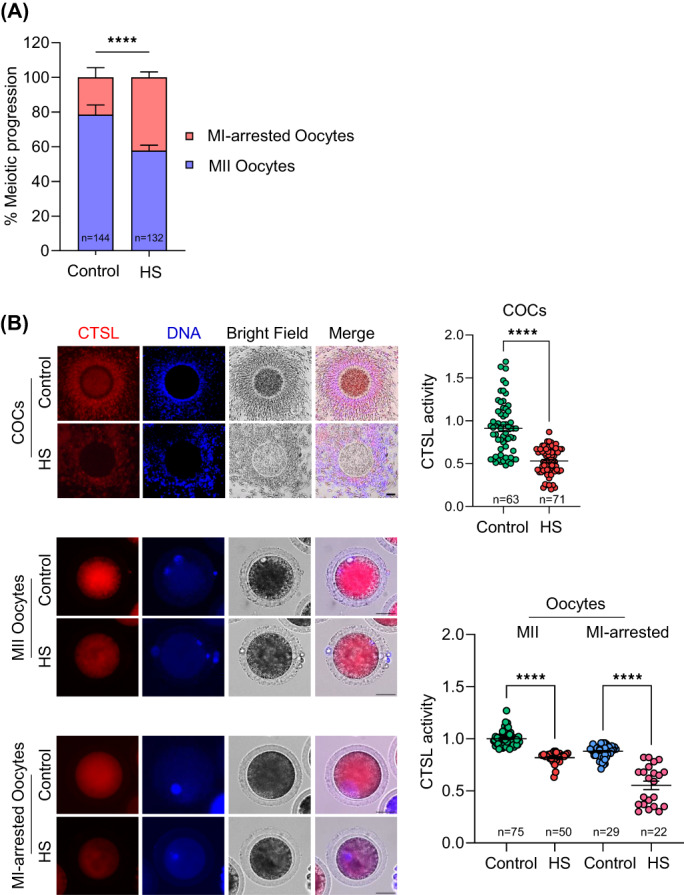
Heat shock perturbs CTSL activity in COCs/oocytes. Bovine COCs were in vitro matured at either 38.5°C (control group) or 41°C (HS group) for 22–24 h followed by quantification of oocytes meiotic progression (A) and detection of intracellular CTSL activity in COCs/denuded oocytes (B). CTSL activity represents the mean values of fluorescent intensity in the indicated cells. Scale bar represents 50 μm. The data of three independent experiments are presented as mean ± SEM. The total number of analysed COCs/oocytes is specified in each graph. Asterisks denote a significant variance (** means *p* < 0.01, and **** means *p* < 0.0001) between the different groups when compared using either Student's *t*‐test (A) or Chi‐square contingency test (B). The number of replicates was included in our statistical model; there was no significant variance between the different replicates. COCs, cumulus‐oocyte complexes; CTSL, cathepsin L; HS, heat shock; SEM, standard error of the mean.

It is well known that the exposure of bovine oocytes to elevated temperatures, either in vivo or in vitro, disrupts their quality and developmental competence.^36^, ^42^, ^43^ Indeed, the exposure of COCs to HS during IVM (22–24 h) resulted in a lower percentage of oocytes reaching MII stage when compared to COCs in vitro matured at the homeothermic temperature of cows (*p* < 0.01; Figure 2A). We employed an in vitro HS model to confirm the association between oocyte quality and CTSL activity. Due to the variance observed between the different oocyte meiotic stages (Figure 1C), CTSL activities were compared between the corresponding stages of control and HS oocytes. As expected, HS during IVM resulted in a significant reduction in CTSL activity in both COCs and oocytes (*p* < 0.0001; Figure 2B).

### 3.2 CTSL inhibition during IVM disturbs oocyte development and compromises the quality of produced embryos

Because our results suggest that CTSL associates with proper oocyte maturation, we then explored the possible involvement of active CTSL in the capacity of oocytes to be fertilized and undergo normal early embryo development. In order to test this, we assessed oocyte developmental competence after CTSL inhibition using LF. LF, through its carboxyl‐terminal lobe, selectively binds to CTSL active site cleft, thereby inhibiting CTSL activity.^44^, ^45^, ^46^ Accordingly, LF was added to IVM medium at different concentrations; we identified that a concentration of 1000 μg/mL causes a significant reduction of CTSL activity in COCs (*p* < 0.0001; Figure S1A). On the other hand, the intracellular activity of other cysteine cathepsins (CTSB and CTSK) in COCs showed no significant difference in the absence or presence of LF in IVM medium (Figure S1B) indicating that both LF and CTSL activity detection assay are specific and efficient to inhibit and detect CTSL, respectively. To exclude the possibility of drug cytotoxicity, LF‐treated COCs were assessed for their viability using PI staining. There was no significant difference in the percentage of dead cells in LF‐treated COCs when compared to non‐treated COCs (Figure S1C).

**FIGURE 3:**
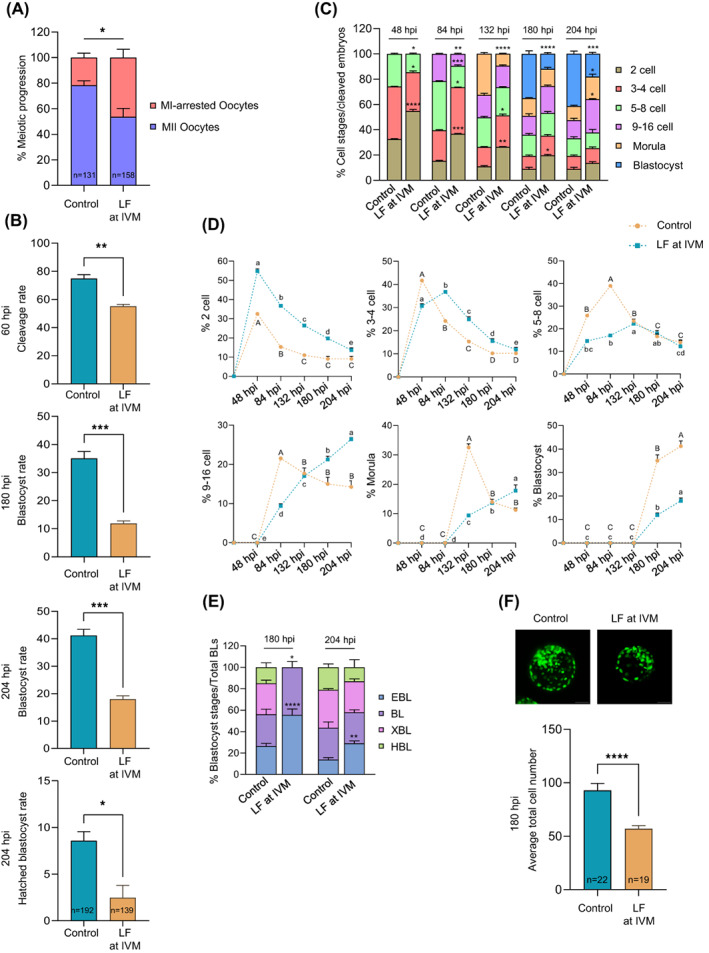
Inhibition of CTSL activity during oocyte maturation results in impaired early embryo development. Bovine COCs were in vitro matured without‐ (control group) or with 1000 μg/mL LF (LF group) followed by the normal IVF and IVC procedures. The data of three independent experiments are presented as mean ± SEM. The total number of analysed oocytes/embryos is specified in each graph. (A) Quantification of oocyte meiotic progression; asterisks denote a significant variance (* means *p* < 0.05) between both groups when compared using Chi‐square contingency test. (B) Assessment of cleavage, blastocyst and HBL rates; asterisks denote a significant variance (* means *p* < 0.05, ** means *p* < 0.01 and *** means *p* < 0.001) between both groups when compared using Student's *t*‐test. (C–E) Quantification of embryonic cell progression in relation to the total number of cleaved embryos (*n* = 144 for control and *n* = 76 for LF). Asterisks in stacked bar graphs (C & E) denote a significant variance (* means *p* < 0.05, ** means *p* < 0.01 and *** means *p* < 0.001) between the corresponding stages of control and LF groups at each time point when compared using Fisher's exact test. Different letters in line graphs (D) denote a significant variance between the successive time points within each cell stage in the same group (i.e., capital letters refer to control and small letters refer to LF) when compared using one‐way ANOVA followed by Tukey's multiple comparisons test. (F) Evaluation of blastocyst total cell number; asterisks denote a significant variance (**** means *p* < 0.0001) between both groups when compared using Student's *t*‐test. COCs, cumulus‐oocyte complexes; CTSL, cathepsin L; HBL, hatched blastocyst; IVC, in vitro culture; IVF, in vitro fertilization; LF, lactoferrin; SEM, standard error of the mean.

Next, COCs were in vitro matured in the absence (control) or presence of LF (1000 μg/mL) for 22–24 h followed by IVF and IVC. Our results showed that LF supplementation during IVM decreased the percentage of oocytes that reach the MII stage (*p* < 0.05; Figure 3A) and embryo cleavage rate after IVF (*p* < 0.05; Figure 3B). In fact, cleaved embryos derived from LF‐treated COCs showed a significant reduction in blastocyst (*p* < 0.001) and HBL (*p* < 0.05) rates when compared to those derived from the non‐treated control COCs (Figure 3B). Of note, the blastocyst and HBL rates were calculated in relation to the number of embryos that reached at least the 2‐cell stage at 60 hpi (i.e., cleavage rate) to eliminate the possibility that the poor developmental competence of LF‐treated COCs was a reflection of oocyte maturation failure.

The above results indicate that low levels of CTSL in COCs/oocytes is associated with poor potency of zygotes to develop into blastocysts. To better understand this phenomenon, we did a detailed characterization of early embryonic development by assessing the number of embryonic cells (i.e., the 2 cell, 3–4 cell, 5–8 cell, 9–16 cell, morula and blastocyst) at specific time points post‐insemination (i.e., 48, 84, 132, 180 and 204 hpi). We observed a delay in the cleavage speed of embryos derived from LF‐treated COCs (i.e., LF group) when compared to those derived from the non‐treated COCs (i.e., control group; Figure 3C). In the control group, the majority of 3–4 cell, 5–8 cell, 9–16 cell and morula stage embryos was recorded at 48, 84, 84 and 132 hpi, respectively, while their counterparts in LF group peaked at 84, 132, 204 and 204 hpi, respectively (Figure 3D). Moreover, a further analysis of the blastocyst components (i.e., EBL, BL, XBL and HBL) at 180 and 204 hpi showed a dissimilar composition between the groups; the proportion of EBL (*p* < 0.001) and BL (*p* < 0.01) was significantly higher in LF group than the control group, and neither XBL nor HBL were observed in LF group at 180 hpi. Further, at 204 hpi, the proportion of EBL was higher (*p* < 0.001) in LF group when compared to controls, indicating a delay in the speed of embryo development (Figure 3E). In line with the poor development observed in the LF group, our analysis revealed that these blastocysts had significantly lower total cell numbers than the control embryos (*p* < 0.0001; Figure 3F).

### 3.3 Supplementation of rCTSL during IVM improves oocyte development under normal and HS conditions

**FIGURE 4:**
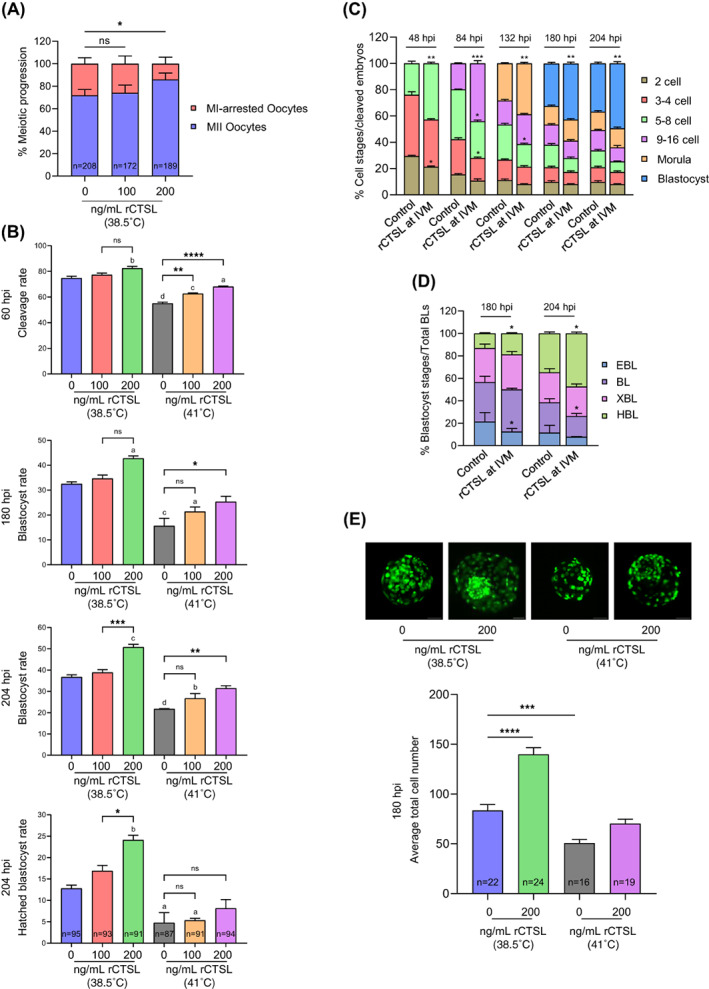
Upregulating CTSL activities in COCs/oocytes enhances their developmental competence under normal and heat shock conditions. Bovine COCs were in vitro matured with rCTSL (0, 100 or 200 ng/mL) at either 38.5°C (control groups) or 41°C (HS groups) for 22–24 h followed by the normal IVF and IVC procedures. The data of three independent experiments are presented as mean ± SEM. The total number of analysed oocytes/embryos is specified in each graph. (A) Quantification of oocyte meiotic progression; asterisks denote a significant variance (* means *p* < 0.05) between both groups when compared using Chi‐square contingency test. (B) Assessment of cleavage, blastocyst and HBL rates. The data were analysed using one‐way ANOVA followed by Tukey's multiple comparisons test. Different letters denote a significant variance ([A] means *p* < 0.05, [B] means *p* < 0.01, [C] means *p* < 0.001 and [D] means *p* < 0.0001) between each group when compared to the control group (COCs in vitro matured at 38.5°C with 0 ng/mL rCTSL), while asterisks denote a significant variance (* means *p* < 0.05, ** means *p* < 0.01, *** means *p* < 0.001 and **** means *p* < 0.0001) between the indicated groups. (C, D) Quantification of embryo cell progression in relation to the total number of cleaved embryos (*n* = 71 for control and *n* = 75 for rCTSL). Asterisks denote a significant variance (* means *p* < 0.05, ** means *p* < 0.01 and *** means *p* < 0.001) between the corresponding stages of control and rCTSL groups at each time‐point when compared using Fisher's exact test. (E) Evaluation of blastocyst total cell number; asterisks denote a significant variance (*** means *p* < 0.001 and **** means *p* < 0.0001) between each group when compared to the control group (COCs in vitro matured at 38.5°C with 0 ng/mL rCTSL) using one‐way ANOVA followed by Tukey's multiple comparisons test. COCs, cumulus‐oocyte complexes; CTSL, cathepsin L; HBL, hatched blastocyst; HS, heat shock; IVC, in vitro culture; IVF, in vitro fertilization; rCTSL, recombinant CTSL; SEM, standard error of the mean.

Because CTSL activity is lower in poor quality versus good quality oocytes and its inhibition perturbs oocyte developmental competence, we asked whether the enhancement of intracellular active CTSL in COCs/oocytes could improve oocyte developmental competence. To this end, rCTSL was added to the IVM medium at different concentrations followed by assessing CTSL activity. Our results indicate that concentrations of 100 and 200 ng/mL, but not 400 ng/mL (likely compromises cell viability), significantly increased CTSL activity in COCs (*p* < 0.0001; Figure S2). Accordingly, we supplemented the IVM medium with 100 or 200 ng/mL rCTSL followed by IVF and IVC; a non‐supplemented group served as control. We observed a significant increase in the percentage of MII oocytes upon supplementation of IVM medium with 200 ng/mL rCTSL (*p* < 0.05; Figure 4A). Further, increasing CSTL levels during oocyte maturation improved embryo development post‐fertilization (*p* < 0.05; Figure 4B). Moreover, embryos originating from rCTSL‐treated COCs showed faster cell progression than those from the non‐treated COCs (Figure 4C), which resulted in a higher percentage of HBL (recorded at 180 and 204 hpi; *p* < 0.01; Figure 4D). In addition, the blastocysts produced from rCTSL group had more cells than those produced from the control group (*p* < 0.0001; Figure 4E). Given the improving effect of rCTSL on oocyte developmental competence, we asked whether rCTSL supplementation during IVM can improve the developmental competence of HS‐exposed COCs which are associated with poor quality and developmental competence (Figure 4B). Accordingly, a group of COCs was exposed to HS during IVM (22–24 h) in the absence or presence of rCTSL followed by IVF and IVC. Our results showed that increasing the levels of CSTL ameliorates the adverse effects of HS on oocyte developmental competence. Indeed, increasing CSTL levels during oocyte maturation of HS‐exposed COCs significantly improved the developmental competence (i.e., increased cleavage and blastocyst rates) of the produced embryos post‐fertilization (Figure 4B) and tended to increase (*p* = 0.058) the average total cell number of the produced blastocysts (Figure 4E).

### 3.4 The activity of CTSL correlates with the development and quality of preimplantation embryos

**FIGURE 5:**
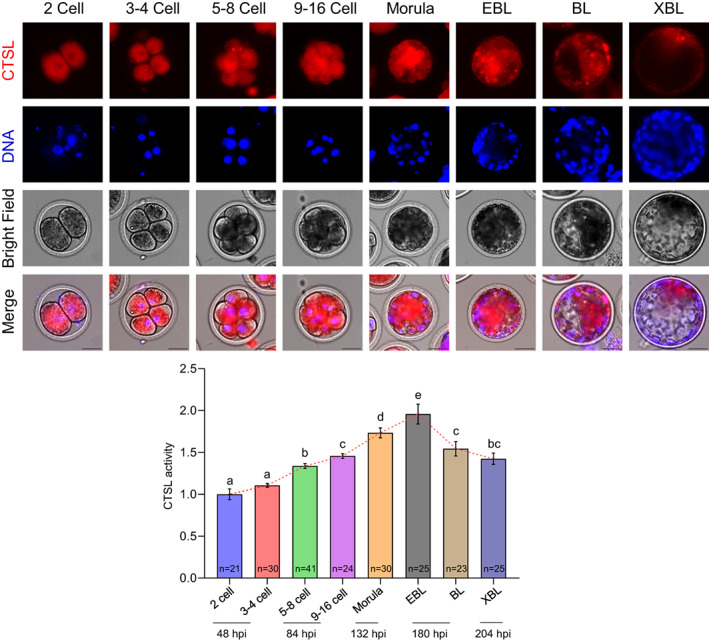
CTSL activity in relation to early embryo development. Detection of intracellular CTSL activity in the developing embryos at the indicated time‐points during IVC period. Scale bar represents 50 μm. The data of three independent experiments are presented as mean ± SEM. The total number of analysed embryos is specified in each cell stage bar. CTSL activity represents the raw integrated density values of its fluorescence intensity in the defined cells. These values were normalized against those of the two cell‐stage embryos. Different letters denote a significant variance (*p* < 0.05) between the successive embryonic stages when compared using one‐way ANOVA followed by Tukey's multiple comparisons test. The number of replicates was included in our statistical model; there was a significant variance (*p* = 0.0001) between the different replicates. CTSL, cathepsin L; IVC, in vitro culture; SEM, standard error of the mean.

Our data strongly support the notion that intracellular CTSL activity in COCs plays an important role in acquiring the oocyte developmental competence. Therefore, we wondered if CTSL would also play a role during early embryonic development. We first measured CTSL activity in preimplantation embryos and found a positive correlation (*r* = 0.69, *p* < 0.001) between CTSL activity and embryo developmental stage up to the EBL stage, with a decline thereafter (Figure 5). Of note, CTSL activity was detected at the time‐points at which each embryo developmental stage reaches its peak (Figure 3C), and the values of CTSL fluorescent intensities for the successive embryonic stages were normalized against those of the 2 cell‐stage embryos (i.e., our starting point).

**FIGURE 6:**
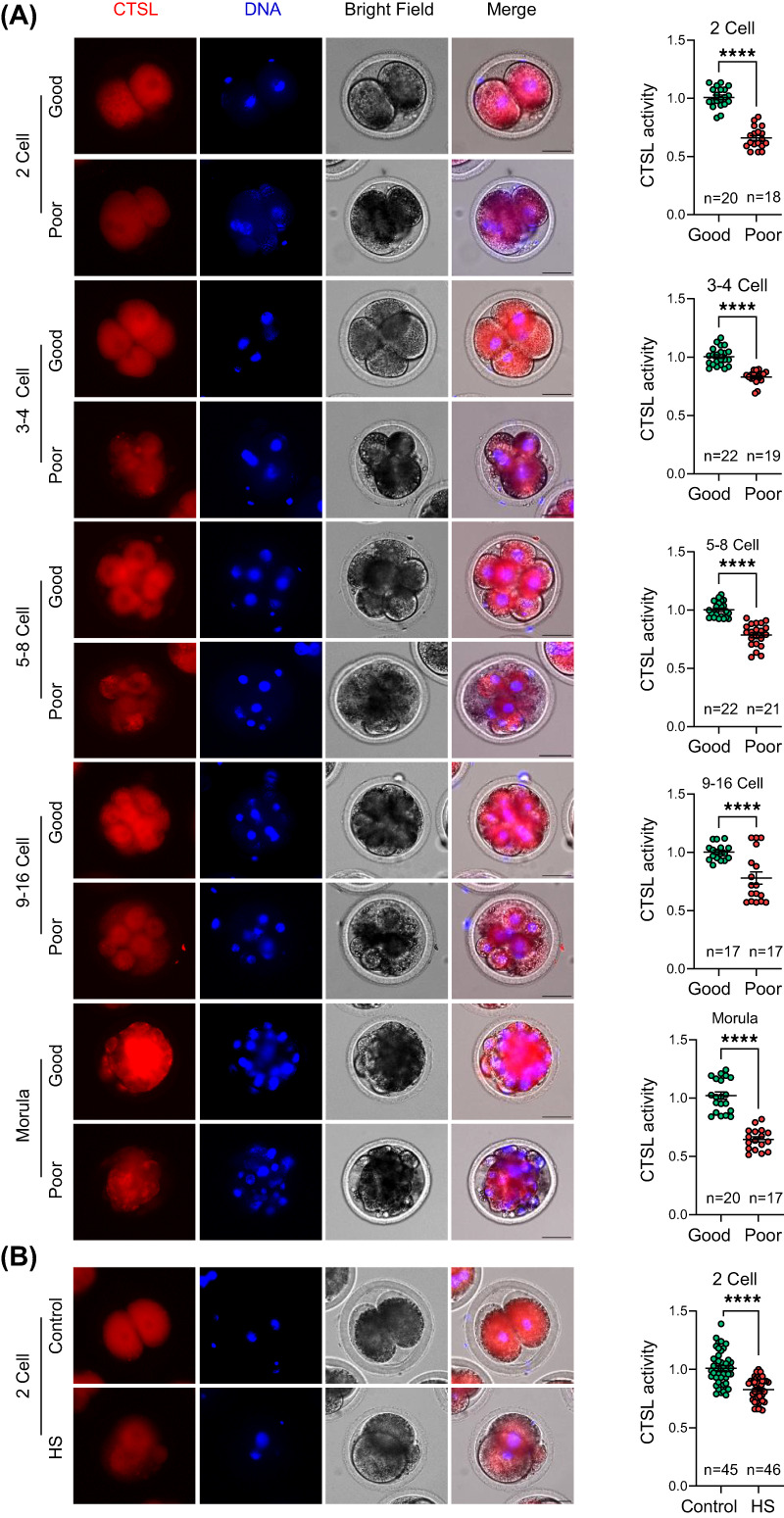
CTSL activity is related to early embryo quality. (A) Detection of intracellular CTSL activity in good and poor quality cleaved embryos. (B) Upon conducting the normal IVM and IVF procedures, the putative zygotes were in vitro cultured at either 38.5°C (control group) or 41°C (HS group) for 12 h followed by the detection of intracellular CTSL activity in the developing embryos. CTSL activity represents the mean values of its fluorescence intensity in the defined cells. Scale bar represents 50 μm. The data of three independent experiments are presented as mean ± SEM. The total number of analysed embryos is specified in each graph. Asterisks denote a significant variance (*** means *p* < 0.001 and **** means *p* < 0.0001) between the different groups when compared using Student's *t*‐test. The number of replicates was included in our statistical model; there was a significant variance (*p* = 0.0001) between the different replicates. The number of replicates was included in our statistical model; there was no significant variance between the different replicates. CTSL, cathepsin L; HS, heat shock; IVF, in vitro fertilization; IVM, in vitro maturation; SEM, standard error of the mean.

The quality of early embryos is largely determined on the basis of cleavage speed and their morphology.^47^, ^48^ Because of the variance in CTSL activity between successive cleavage‐stage embryos identified here, we examined embryo morphology to ascribe embryo quality. The embryo morphology assessment routinely evaluates the cytoplasmic appearance, size and symmetry of blastomeres, degree of fragmentation, presence of vacuoles and the thickness of zona pellucida.^49^ To investigate if there is an association between embryo morphology and CTSL activity, we assayed CTSL activity in embryos of similar stages at specific time‐points after insemination. We observed that the embryos showing morphological abnormalities (e.g., low cytoplasmic homogeneity, asymmetrical blastomeres, high degree of fragmentation or vacuoles) had lower CTSL activity (*p* < 0.001; Figure 6A). Our previously demonstrated that applying HS to early preimplantation embryos alters embryo morphology in several ways with a subsequent impairment of embryo quality and developmental competence.^50^, ^51^, ^52^ To ensure that the lower CTSL activity in morphologically‐abnormal embryos reflects their lower quality, we exposed putative zygotes ~12 h after insemination to a physiologically relevant heat shock^37^ during the first 12 h of IVC followed by assessing CTSL activity. Expectedly, CTSL activity was significantly lower in HS‐exposed embryos (i.e., poor quality embryos) when compared to controls (*p* < 0.0001; Figure 6B).

### 3.5 CTSL inhibition during IVC perturbs the development and quality of early embryos

**FIGURE 7:**
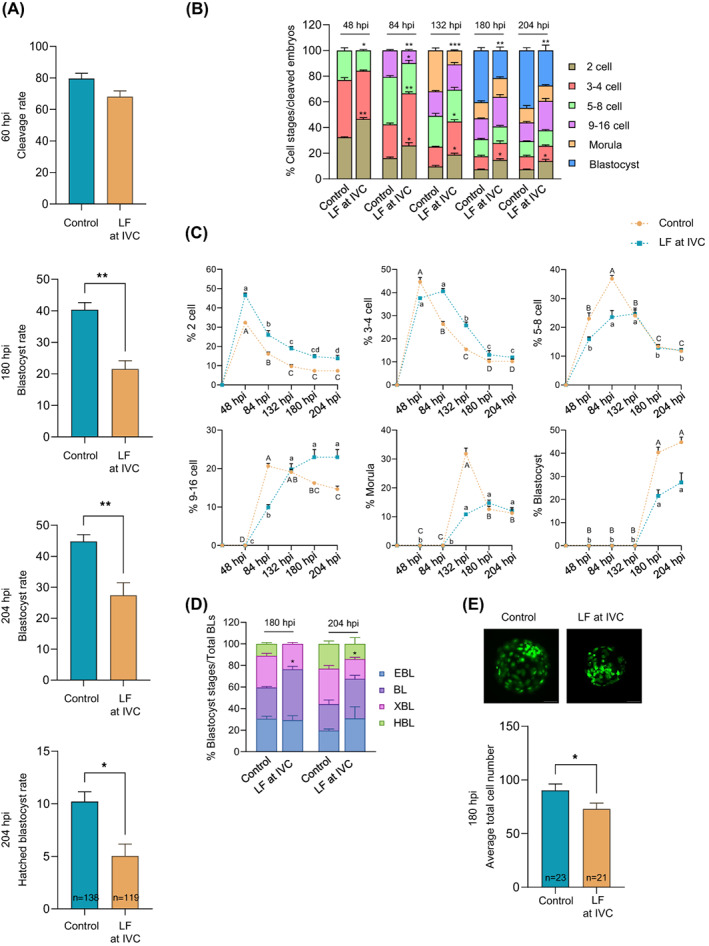
Inhibition of CTSL activity in early embryos impairs their development and quality. Upon conducting the normal IVM and IVF procedures, the putative zygotes were in vitro cultured without (control group) or with 1000 μg/mL LF (LF group) for 7–8 days. The data of three independent experiments are presented as mean ± SEM. The total number of analysed embryos is specified in each graph. (A) Assessment of cleavage, blastocyst and HBL rates; asterisks denote a significant variance (* means *p* < 0.05, and ** means *p* < 0.01) between both groups when compared using Student's *t*‐test. (B–D) Quantification of embryonic cell progression in relation to the total number of cleaved embryos (*n* = 111 for control and *n* = 83 for LF). Asterisks in stacked bar graphs (B & D) denote a significant variance (* means *p* < 0.05, ** means *p* < 0.01 and *** means *p* < 0.001) between the corresponding stages of control and LF groups at each time‐point when compared using Fisher's exact test. Different letters in line graphs (C) denote a significant variance between the successive time‐points within each cell stage in the same group (i.e., capital letters refer to control and small letters refer to LF) when compared using one‐way ANOVA followed by Tukey's multiple comparisons test. (E) Evaluation of blastocyst total cell number; asterisks denote a significant variance (* means *p* < 0.05) between both groups when compared using Student's *t*‐test. CTSL, cathepsin L; HBL, hatched blastocyst; IVF, in vitro fertilization; IVM, in vitro maturation; LF, lactoferrin; SEM, standard error of the mean.

Linking the embryo quality to intracellular CTSL activity led us to hypothesize that CTSL is involved in regulating early embryo development. To test our hypothesis, we first investigated the effect of CTSL inhibition on embryo development. Putative zygotes were in vitro cultured in the absence (control) or presence of LF (1000 μg/mL) for 7–8 days. Our data revealed that LF‐treated zygotes have a lower developmental competence when compared to non‐treated zygotes (*p* < 0.05; Figure 7A). It should be emphasized that 1000 μg/mL LF supplementation to IVC medium has the capability of inhibiting CTSL (*p* < 0.0001; Figure S3A). Moreover, there was no significant difference in the percentage of dead cells in LF‐treated embryos when compared to non‐treated embryos (Figure S3B).

To uncover how the reduction of intracellular CTSL activity negatively affects early embryo development, we monitored cleavage progression at specific time points post‐insemination. There was a delay in embryonic cell progression upon CTSL reduction (Figure 7B,C); such delay was highly apparent by the complete lack of HBL at 180 hpi in LF group (Figure 7D). Moreover, we found reduced total cell numbers in blastocysts from the LF group when compared to the control group (*p* < 0.05; Figure 7E).

### 3.6 Supplementation of rCTSL during IVC improves the development and quality of early embryos under normal and HS conditions

**FIGURE 8:**
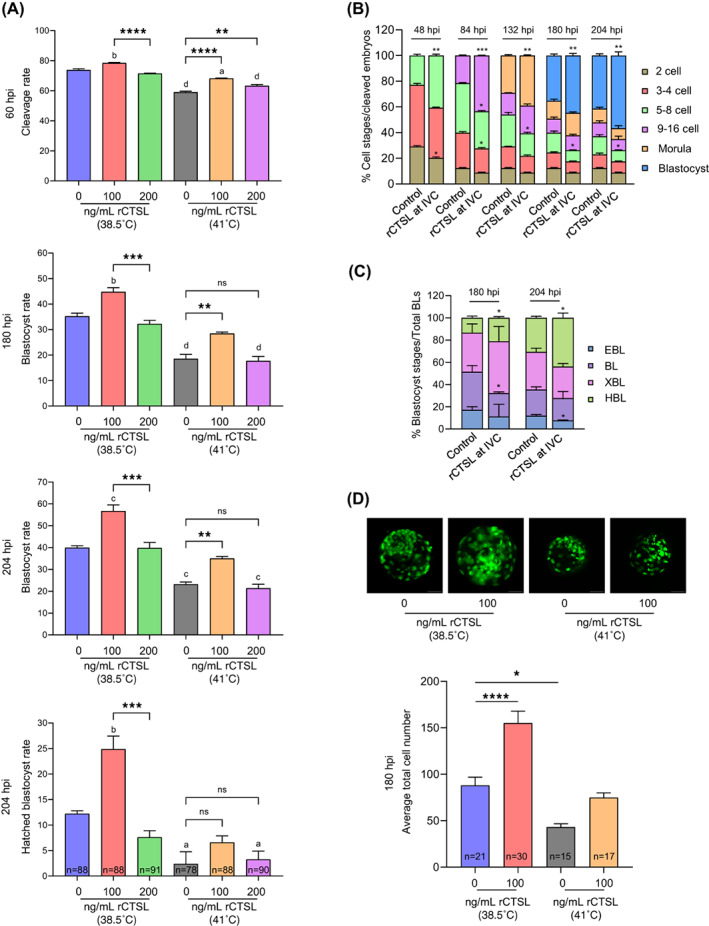
Enhancing CTSL activities in early embryos improves their development under normal and heat shock conditions. The putative zygotes were in vitro cultured with rCTSL (0, 100 or 200 ng/mL) at either 38.5°C (control groups) or 41°C (HS groups) for 7–8 days. The data of three independent experiments are presented as mean ± SEM. The total number of analysed embryos is specified in each graph. (A) Assessment of cleavage, blastocyst and HBL rates. The data were analysed using one‐way ANOVA with Tukey's multiple comparisons test. Different letters denote a significant variance ([a] means *p* < 0.05, [b] means *p* < 0.01, [c] means *p* < 0.001 and [d] means *p* < 0.0001) between each group when compared to the control group (zygotes in vitro cultured at 38.5°C with 0 ng/mL rCTSL), while asterisks denote a significant variance (** means *p* < 0.01 and *** means *p* < 0.001) between the indicated groups. (B, C) Quantification of embryonic cell progression in relation to the total number of cleaved embryos (*n* = 65 for control and *n* = 69 for rCTSL). Asterisks denote a significant variance (* means *p* < 0.05, ** means *p* < 0.01 and *** means *p* < 0.001) between the corresponding stages of control and rCTSL groups at each time‐point when compared using Fisher's exact test. (D) Evaluation of blastocyst total cell number; asterisks denote a significant variance (* means *p* < 0.05 and **** means *p* < 0.0001) between each group when compared to the control group (zygotes in vitro cultured at 38.5°C with 0 ng/mL rCTSL) using one‐way ANOVA followed by Tukey's multiple comparisons test. CTSL, cathepsin L; HBL, hatched blastocyst; HS, heat shock; rCTSL, recombinant CTSL; SEM, standard error of the mean.

Our data show that reduced CTSL activity compromises embryonic development. To investigate whether enhancing CTSL activity would improve embryo development, putative zygotes were in vitro cultured in the absence (control) or presence of rCTSL (100 or 200 ng/mL) for 7–8 days. Supplementation of the culture medium with rCTSL significantly increased CTSL activity when measured at the 2‐cell stage (*p* < 0.0001; Figure S4). We found that 100 ng/mL rCTSL supplementation improved embryo development while supplementing the medium with 200 ng/mL had no effect on embryo development (Figure 8A). Data also demonstrated that enhanced CTSL activity accelerates the embryo development (Figure 8B). A thorough analysis of the blastocysts derived from rCTSL‐treated zygotes revealed a higher proportion of HBL recorded at 180 and 204 hpi in the expense of BL and EBL, respectively (*p* < 0.05; Figure 8C). Furthermore, the blastocyst derived from rCTSL‐treated zygotes showed higher total cell number than those derived from the non‐treated zygotes (*p* < 0.001; Figure 8D). Concurrently, another group of putative zygotes was exposed to 41°C for the first 12 h of IVC (in the absence or presence of rCTSL), then returned to control temperature (38.5°C) for the remainder of the IVC period. Results showed that the rCTSL supplementation to the IVC medium (at 100 ng/mL) significantly improved the developmental competence of HS‐exposed embryos (Figure 8A) and tended to increase (*p* = 0.12) the average total cell number of the produced blastocysts (Figure 8D) when compared to non‐treated HS‐exposed blastocysts.

## 4 DISCUSSION

The present study, using a bovine oocyte/embryo model, provides novel evidence that CTSL plays a pivotal role in regulating oocyte meiosis and preimplantation embryo development. We show that the depletion of CTSL activity in the oocytes or zygotes has a negative impact on their quality and, in turn, their developmental competence. In addition, our results demonstrate that upregulating CTSL activity in the oocytes or zygotes shows great potential in improving the quality of preimplantation mammalian embryos with clear applied implications for enhancing assisted reproductive technologies.

Our findings demonstrate a positive correlation between CTSL activity and mammalian preimplantation embryo development. Consistent with our results, monitoring the activity of CTSL protease in zebrafish embryos from the start of fertilization until hatching revealed a time‐dependent increase in CTSL activity during the entire process of embryogenesis.^53^ Moreover, CTSL is required for *Caenorhabditis elegans* embryo development and its disruption results in the accumulation of yolk platelets in the cytoplasm of early embryos, leading to a high percentage of arrested non‐viable embryos and decelerated progression of viable embryos.^54^, ^55^ Several potential mechanisms can explain the pivotal role of CTSL in early embryo development. Given that protein and amino acid turnover is essential during oocyte maturation^56^ and early embryo development,^57^, ^58^ it is possible that intracellular active CTSL, as a cysteine protease, is involved in the regulation of protein degradation and synthesis, which are necessary for oocyte and embryo development and quality. For example, CTSL is essential for the degradation of the inherited sperm histones in sea urchin embryos, a process required for the initiation of cell cleavage.^59^, ^60^ Consequently, interfering with CTSL function prevents the post‐fertilization degradation of these histones and perturbed early embryo development.^61^, ^62^ Additionally, CTSL can also induce autophagy in a variety of cell types.^63^, ^64^, ^65^, ^66^ Since autophagy is required for oocyte meiosis and embryo development under normal and HS conditions,^20^, ^67^, ^68^, ^69^, ^70^, ^71^, ^72^, ^73^, ^74^, ^75^ it is plausible that CTSL regulates oocyte and embryo development via autophagy activation. These mechanisms (i.e., regulating the protein turnover machinery and autophagy) may also explain why CTSL upregulation in oocytes and early embryos protects, at least partially, against the adverse effects of HS. However, further research is necessary to fully comprehend the underlying molecular mechanisms underpinning the involvement of CTSL in regulating embryo quality and enhancing its tolerance to adverse conditions.

We previously demonstrated that the activities of cysteine proteases, CTSB and CTSK, are inversely correlated with oocyte and embryo developmental competence.^20^, ^23^, ^24^ In a stark contrast to CTSB and CTSK, we found that CTSL activity correlates with oocyte and embryo developmental competence. Although lysosomal cysteine proteases are involved in the breakdown of a wide range of intracellular and extracellular proteins,^16^ cathepsins may differ in their substrate specificities and their roles in physiological and pathological processes.^17^, ^18^ For example, CTSB is involved in the degradation of extracellular matrix proteins (e.g., collagen, elastin and fibronectin)^76^ and its proteolytic activity (at its normal physiological level) is essential for several cellular functions.^77^ However, excessive CTSB activity is detrimental for both oocyte and early embryo development in cattle,^23^, ^24^, ^25^ sheep^78^, ^79^ and pigs.^80^ On the contrary, CTSL has a wider range of substrates than CTSB^81^ and has been implicated in several physiological and pathological processes including bone remodelling, angiogenesis and tumour invasion.^82^ Moreover, CTSL has been identified as an important embryotrophin in bovine preimplantation embryos.^83^ Determining the target substrates of the aforementioned cathepsins is essential to understand how these cathepsins differ in their roles in oocyte and early embryo development.

While our data provide several lines of evidence that the intracellular CTSL activity is a prerequisite for the acquisition of oocyte developmental competence, we did not reveal whether such beneficial effect of CTSL is attributed to its activity in oocytes themselves and/or their surrounding cumulus cells. A bi‐directional communication exists between the oocyte and cumulus cells throughout folliculogenesis and oogenesis^84^; such bi‐directional communication allows the continuous flow of essential nutrients and signals between the oocyte and its surrounding cumulus cells and thereby enhancing their functions.^85^, ^86^ Therefore, further investigation is required to address this question.

Reducing CTSL activity by LF treatment in oocytes and zygotes disturbs early embryo development. These findings are in accordance with previous observations that using LF, to inhibit bovine herpesvirus 1, during embryo culture is accompanied by embryo developmental suppression.^87^ Although LF is a selective inhibitor of CTSL,^44^, ^45^ it can also promote CTSG activity.^88^ However, RNA sequencing of bovine oocytes and embryos at different developmental stages revealed that CTSG gene is not expressed in either oocytes or embryos.^89^, ^90^, ^91^, ^92^ Moreover, our observations revealed that LF does not affect CTSB or CTSK, the only cathepsins identified to play roles in bovine oocytes and embryos. Furthermore, enhancing CTSL activity using rCTSL improved oocyte and early embryo development. Taken together, our results suggest that LF impairs oocyte and embryo development by inhibiting CTSL activity.

Lactoferrin is expressed in the mucosal epithelium of the reproductive organs and is also detected in seminal plasma, oviductal, uterine, cervical and vaginal fluids.^93^ A recent study identified a negative correlation between LF levels in the cervical fluid and IVF success rates in women; such negative correlation was highly apparent when the age of patient was ≥35 years.^94^ It is well established that oocytes from females of advanced reproductive age are of lower quality than those of younger females.^95^ Therefore, it is tempting to speculate that LF accumulation in genital tract secretions of women with advanced reproductive age, above its physiological levels, contributes to age‐associated reduced oocyte/embryo quality, at least in part, by inhibiting intracellular CTSL activity in oocytes and zygotes.

## AUTHOR CONTRIBUTIONS

**Mohamed Ezz:** Methodology; investigation; formal analysis; writing—original draft; writing—review & editing. **Masashi Takahashi:** Methodology; resources; writing—review & editing. **Rocio Rivera:** Conceptualization; methodology; formal analysis; resources; writing—review & editing. supervision; project administration; funding acquisition. **Ahmed Balboula:** Conceptualization; methodology; resources; writing—review & editing. supervision; project administration; funding acquisition.

## CONFLICT OF INTEREST STATEMENT

The authors declare that they have no conflict of interest.

## Supporting information


**FIGURE S1:** Lactoferrin inhibits CTSL activity in COCs without disturbing cell livability. Bovine COCs were in vitro matured with LF (at 0, 10, 100 or 1000 μg/mL) for 22–24 h followed by detecting intracellular CTSL activity (A), CTSB/K activity (B) or the assessing the cell livability (B) in LF‐treated COCs. Positive‐control COCs were treated with DNase (50 U/mL) for 1 h prior to PI/Hoechst 33342 staining, while negative‐control COCs were stained with Hoechst 33342 (PI staining was excluded), Scale bar represents 50 μm. The data of three independent experiments are presented as mean ± SEM. The total number of analysed COCs was specified in each graph. Asterisks denote a significant variance (**** means *p* < 0.0001) between the different groups when compared using one‐way ANOVA followed by Tukey's multiple comparisons test. The number of replicates was included in our statistical model; there was no significant variance between the different replicates.
**FIGURE S2:** Recombinant human CTSL enhances CTSL activity in COCs. Bovine COCs were in vitro matured with rCTSL (at 0, 100, 200 or 400 ng/mL) for 22–24 h followed by the detection of intracellular CTSL activity. Scale bar represents 50 μm. The data of three independent experiments are presented as mean ± SEM. The total number of analysed COCs was specified in each graph. Asterisks denote a significant variance (** means *p* < 0.01, and **** means *p* < 0.0001) between the different groups when compared using one‐way ANOVA followed by Tukey's multiple comparisons test. The number of replicates was included in our statistical model; there was no significant variance between the different replicates.
**FIGURE S3:** Lactoferrin inhibits CTSL activity in early embryos without disturbing cell livability. Cleaved embryos at 60 hpi were in vitro cultured with LF (at 0 or 1000 μg/mL) for 24 h, followed by the detection of intracellular CTSL activity (A) or the assessment of cell livability (B) in LF‐treated embryos. Positive‐control embryos were treated with DNase (50 U/mL) for 1 h prior to PI/Hoechst 33342 staining, while negative‐control embryos were stained with Hoechst 33342 (PI staining was excluded). Scale bar represents 50 μm. The data of three independent experiments are presented as mean ± SEM. The total number of analysed embryos was specified in each graph. Asterisks denote a significant variance (**** means *p* < 0.0001) between the different groups when compared using either Student's *t*‐test (A) or one‐way ANOVA followed by Tukey's multiple comparisons test (B). The number of replicates was included in our statistical model; there was a significant variance (*p* = 0.0046) between the different replicates (A).
**FIGURE S4:** Recombinant human CTSL enhances CTSL activity in early embryos. Upon conducting the normal IVM and IVF procedures, the putative zygotes were in vitro cultured with rCTSL (at 0, 100 or 200 ng/mL) for 24 h followed by the detection of intracellular CTSL activity. Scale bar represents 50 μm. The data of three independent experiments are presented as mean ± SEM. The total number of analysed embryos was specified in the graph. Asterisks denote a significant variance (**** means *p* < 0.0001) between the different groups when compared using one‐way ANOVA followed by Tukey's multiple comparisons test. The number of replicates was included in our statistical model; there was no significant variance between the different replicates.

## Data Availability

The data that support the findings of this study are available from the corresponding author upon reasonable request.

## References

[1] Benagiano G , Farris M , Grudzinskas G . Fate of fertilized human oocytes. Reprod Biomed Online. 2010;21(6):732‐741. 21050816 10.1016/j.rbmo.2010.08.011

[2] Diskin MG , Morris DG . Embryonic and early foetal losses in cattle and other ruminants. Reprod Domest Anim. 2008;43(Suppl 2):260‐267. 18638133 10.1111/j.1439-0531.2008.01171.x

[3] Wang X , Wu G , Bazer FW . mTOR: the Master Regulator of Conceptus Development in Response to Uterine Histotroph during Pregnancy in Ungulates. 2016.

[4] Bazer FW , First NL . Pregnancy and parturition. J Anim Sci. 1983;57(Suppl 2):425‐460. 6352591

[5] Simon A , Laufer N . Assessment and treatment of repeated implantation failure (RIF). J Assist Reprod Genet. 2012;29(11):1227‐1239. 22976427 10.1007/s10815-012-9861-4 PMC3510376

[6] Laux T , Jurgens G . Embryogenesis: a new start in life. Plant Cell. 1997;9(7):989‐1000. 12237371 10.1105/tpc.9.7.989 PMC156973

[7] Magli MC , Jones GM , Lundin K , van den Abbeel E . Atlas of human embryology: from oocytes to preimplantation embryos. Preface. Hum Reprod. 2012;27(Suppl 1):i1. 22863770 10.1093/humrep/des229

[8] Cobo A , Coello A , Remohi J , Serrano J , de Los Santos JM , Meseguer M . Effect of oocyte vitrification on embryo quality: time‐lapse analysis and morphokinetic evaluation. Fertil Steril. 2017;108(3):491‐497 e493. 28865549 10.1016/j.fertnstert.2017.06.024

[9] Demko ZP , Simon AL , McCoy RC , Petrov DA , Rabinowitz M . Effects of maternal age on euploidy rates in a large cohort of embryos analyzed with 24‐chromosome single‐nucleotide polymorphism‐based preimplantation genetic screening. Fertil Steril. 2016;105(5):1307‐1313. 26868992 10.1016/j.fertnstert.2016.01.025

[10] Lazzaroni‐Tealdi E , Barad DH , Albertini DF , et al. Oocyte scoring enhances embryo‐scoring in predicting pregnancy chances with IVF where it counts Most. PLoS One. 2015;10(12):e0143632. 26630267 10.1371/journal.pone.0143632 PMC4668065

[11] Parinaud J , Mieusset R , Vieitez G , Labal B , Richoilley G . Influence of sperm parameters on embryo quality. Fertil Steril. 1993;60(5):888‐892. 8224276 10.1016/s0015-0282(16)56292-x

[12] Giorgetti C , Terriou P , Auquier P , et al. Embryo score to predict implantation after in‐vitro fertilization: based on 957 single embryo transfers. Hum Reprod. 1995;10(9):2427‐2431. 8530679 10.1093/oxfordjournals.humrep.a136312

[13] Van Royen E , Mangelschots K , De Neubourg D , et al. Characterization of a top quality embryo, a step towards single‐embryo transfer. Hum Reprod. 1999;14(9):2345‐2349. 10469708 10.1093/humrep/14.9.2345

[14] Ziebe S , Petersen K , Lindenberg S , Andersen AG , Gabrielsen A , Andersen AN . Embryo morphology or cleavage stage: how to select the best embryos for transfer after in‐vitro fertilization. Hum Reprod. 1997;12(7):1545‐1549. 9262293 10.1093/humrep/12.7.1545

[15] Gad A , Hoelker M , Besenfelder U , et al. Molecular mechanisms and pathways involved in bovine embryonic genome activation and their regulation by alternative in vivo and in vitro culture conditions. Biol Reprod. 2012;87(4):100. 22811576 10.1095/biolreprod.112.099697

[16] Vasiljeva O , Reinheckel T , Peters C , Turk D , Turk V , Turk B . Emerging roles of cysteine cathepsins in disease and their potential as drug targets. Curr Pharm Des. 2007;13(4):387‐403. 17311556 10.2174/138161207780162962

[17] Brix K , Dunkhorst A , Mayer K , Jordans S . Cysteine cathepsins: cellular roadmap to different functions. Biochimie. 2008;90(2):194‐207. 17825974 10.1016/j.biochi.2007.07.024

[18] Patel S , Homaei A , El‐Seedi HR , Akhtar N . Cathepsins: proteases that are vital for survival but can also be fatal. Biomed Pharmacother. 2018;105:526‐532. 29885636 10.1016/j.biopha.2018.05.148 PMC7172164

[19] Turk V , Stoka V , Vasiljeva O , et al. Cysteine cathepsins: from structure, function and regulation to new frontiers. Biochim Biophys Acta. 2012;1824(1):68‐88. 22024571 10.1016/j.bbapap.2011.10.002 PMC7105208

[20] Balboula AZ , Aboelenain M , Li J , et al. Inverse relationship between autophagy and CTSK is related to bovine embryo quality. Reproduction. 2020;159(6):757‐766. 32224503 10.1530/REP-20-0036

[21] Balboula AZ , Aboelenain M , Sakatani M , et al. Effect of E‐64 supplementation during in vitro maturation on the developmental competence of bovine OPU‐derived oocytes. Genes (Basel). 2022;13(2):324. 10.3390/genes13020324 PMC8872247 35205369

[22] Balboula AZ , Schindler K , Kotani T , Kawahara M , Takahashi M . Vitrification‐induced activation of lysosomal cathepsin B perturbs spindle assembly checkpoint function in mouse oocytes. Mol Hum Reprod. 2020;26(9):689‐701. 32634244 10.1093/molehr/gaaa051 PMC7828578

[23] Balboula AZ , Yamanaka K , Sakatani M , Hegab AO , Zaabel SM , Takahashi M . Intracellular cathepsin B activity is inversely correlated with the quality and developmental competence of bovine preimplantation embryos. Mol Reprod Dev. 2010;77(12):1031‐1039. 21104746 10.1002/mrd.21250

[24] Balboula AZ , Yamanaka K , Sakatani M , Hegab AO , Zaabel SM , Takahashi M . Cathepsin B activity is related to the quality of bovine cumulus oocyte complexes and its inhibition can improve their developmental competence. Mol Reprod Dev. 2010;77(5):439‐448. 20198711 10.1002/mrd.21164

[25] Balboula AZ , Yamanaka K , Sakatani M , et al. Cathepsin B activity has a crucial role in the developmental competence of bovine cumulus‐oocyte complexes exposed to heat shock during in vitro maturation. Reproduction. 2013;146(4):407‐417. 23898216 10.1530/REP-13-0179

[26] Li J , Balboula AZ , Aboelenain M , et al. Effect of autophagy induction and cathepsin B inhibition on developmental competence of poor quality bovine oocytes. J Reprod Dev. 2020;66(1):83‐91. 31875588 10.1262/jrd.2019-123 PMC7040212

[27] Li J , Maeji M , Balboula AZ , et al. Dynamic status of lysosomal cathepsin in bovine oocytes and preimplantation embryos. J Reprod Dev. 2020;66(1):9‐17. 31685761 10.1262/jrd.2019-115 PMC7040204

[28] Yamanaka KI , Khatun H , Egashira J , et al. Heat‐shock‐induced cathepsin B activity during IVF and culture compromises the developmental competence of bovine embryos. Theriogenology. 2018;114:293‐300. 29677632 10.1016/j.theriogenology.2018.04.005

[29] Duncan EM , Muratore‐Schroeder TL , Cook RG , et al. Cathepsin L proteolytically processes histone H3 during mouse embryonic stem cell differentiation. Cell. 2008;135(2):284‐294. 18957203 10.1016/j.cell.2008.09.055 PMC2579750

[30] Afonso S , Romagnano L , Babiarz B . The expression and function of cystatin C and cathepsin B and cathepsin L during mouse embryo implantation and placentation. Development. 1997;124(17):3415‐3425. 9310336 10.1242/dev.124.17.3415

[31] Song G , Bazer FW , Spencer TE . Differential expression of cathepsins and cystatin C in ovine uteroplacental tissues. Placenta. 2007;28(10):1091‐1098. 17555811 10.1016/j.placenta.2007.04.004

[32] Song G , Spencer TE , Bazer FW . Cathepsins in the ovine uterus: regulation by pregnancy, progesterone, and interferon tau. Endocrinology. 2005;146(11):4825‐4833. 16099855 10.1210/en.2005-0768

[33] Song G , Spencer TE , Bazer FW . Progesterone and interferon‐tau regulate cystatin C in the endometrium. Endocrinology. 2006;147(7):3478‐3483. 16556762 10.1210/en.2006-0122

[34] Chen Z , Robbins KM , Wells KD , Rivera RM . Large offspring syndrome: a bovine model for the human loss‐of‐imprinting overgrowth syndrome Beckwith‐Wiedemann. Epigenetics. 2013;8(6):591‐601. 23751783 10.4161/epi.24655 PMC3857339

[35] Hazeleger NL , Hill DJ , Stubbings RB , Walton JS . Relationship of morphology and follicular‐fluid environment of bovine oocytes to their developmental potential in‐vitro. Theriogenology. 1995;43(2):509‐522. 16727642 10.1016/0093-691x(94)00043-t

[36] Edwards JL , Hansen PJ . Elevated temperature increases heat shock protein 70 synthesis in bovine two‐cell embryos and compromises function of maturing oocytes. Biol Reprod. 1996;55(2):341‐346. 8828838 10.1095/biolreprod55.2.341

[37] Rivera RM , Hansen PJ . Development of cultured bovine embryos after exposure to high temperatures in the physiological range. Reproduction. 2001;121(1):107‐115. 11226033

[38] Huarcaya AP , Drobny S , Marques ARA , et al. Recombinant pro‐CTSD (cathepsin D) enhances SNCA/alpha‐synuclein degradation in alpha‐synucleinopathy models. Autophagy. 2022;18(5):1127‐1151. 35287553 10.1080/15548627.2022.2045534 PMC9196656

[39] Marques ARA , Di Spiezio A , Thiessen N , et al. Enzyme replacement therapy with recombinant pro‐CTSD (cathepsin D) corrects defective proteolysis and autophagy in neuronal ceroid lipofuscinosis. Autophagy. 2020;16(5):811‐825. 31282275 10.1080/15548627.2019.1637200 PMC7158922

[40] Khosravi‐Farsani S , Sobhani A , Amidi F , Mahmoudi R . Mouse oocyte vitrification: the effects of two methods on maturing germinal vesicle breakdown oocytes. J Assist Reprod Genet. 2010;27(5):233‐238. 20407816 10.1007/s10815-010-9411-x PMC2881200

[41] Dominko T , First NL . Timing of meiotic progression in bovine oocytes and its effect on early embryo development. Mol Reprod Dev. 1997;47(4):456‐467. 9211431 10.1002/(SICI)1098-2795(199708)47:4<456::AID-MRD13>3.0.CO;2-U

[42] Lawrence JL , Payton RR , Godkin JD , Saxton AM , Schrick FN , Edwards JL . Retinol improves development of bovine oocytes compromised by heat stress during maturation. J Dairy Sci. 2004;87(8):2449‐2454. 15328267 10.3168/jds.S0022-0302(04)73368-8

[43] Roth Z , Hansen PJ . Disruption of nuclear maturation and rearrangement of cytoskeletal elements in bovine oocytes exposed to heat shock during maturation. Reproduction. 2005;129(2):235‐244. 15695618 10.1530/rep.1.00394

[44] Ohashi A , Murata E , Yamamoto K , et al. New functions of lactoferrin and beta‐casein in mammalian milk as cysteine protease inhibitors. Biochem Biophys Res Commun. 2003;306(1):98‐103. 12788072 10.1016/s0006-291x(03)00917-3

[45] Sano E , Miyauchi R , Takakura N , et al. Cysteine protease inhibitors in various milk preparations and its importance as a food. Food Res Int. 2005;38(4):427‐433.

[46] Madadlou A . Food proteins are a potential resource for mining cathepsin L inhibitory drugs to combat SARS‐CoV‐2. Eur J Pharmacol. 2020;885:173499. 32841639 10.1016/j.ejphar.2020.173499 PMC7443098

[47] Claman P , Armant DR , Seibel MM , Wang TA , Oskowitz SP , Taymor ML . The impact of embryo quality and quantity on implantation and the establishment of viable pregnancies. J In Vitro Fert Embryo Transf. 1987;4(4):218‐222. 10.1007/BF01533759 3625001

[48] Cummins JM , Breen TM , Harrison KL , Shaw JM , Wilson LM , Hennessey JF . A formula for scoring human embryo growth rates in in vitro fertilization: its value in predicting pregnancy and in comparison with visual estimates of embryo quality. J In Vitro Fert Embryo Transf. 1986;3(5):284‐295. 10.1007/BF01133388 3783014

[49] Dennis SJ , Thomas MA , Williams DB , Robins JC . Embryo morphology score on day 3 is predictive of implantation and live birth rates. J Assist Reprod Genet. 2006;23(4):171‐175. 16758347 10.1007/s10815-006-9027-3 PMC3454961

[50] Rivera RM , Kelley KL , Erdos GW , Hansen PJ . Alterations in ultrastructural morphology of two‐cell bovine embryos produced in vitro and in vivo following a physiologically relevant heat shock. Biol Reprod. 2003;69(6):2068‐2077. 12930717 10.1095/biolreprod.103.020347

[51] Rivera RM , Dahlgren GM , Paula LADE , Kennedy RT , Hansen PJ . Actions of thermal stress in two‐cell bovine embryos: oxygen metabolism, glutathione and ATP content, and the time‐course of development. Reproduction. 2004;128(1):33‐42. 15232062 10.1530/rep.1.00146

[52] Rivera RM , Kelley KL , Erdos GW , Hansen PJ . Reorganization of microfilaments and microtubules by thermal stress in two‐cell bovine embryos. Biol Reprod. 2004;70(6):1852‐1862. 14960486 10.1095/biolreprod.103.024901

[53] Kuster E , Kalkhof S , Aulhorn S , von Bergen M , Gundel U . Effects of five substances with different modes of action on Cathepsin H, C and L activities in zebrafish embryos. Int J Environ Res Public Health. 2019;16(20):3956. 10.3390/ijerph16203956 PMC6843663 31627361

[54] Britton C , Murray L . Cathepsin L protease (CPL‐1) is essential for yolk processing during embryogenesis in *Caenorhabditis elegans* . J Cell Sci. 2004;117(Pt 21):5133‐5143. 15456850 10.1242/jcs.01387

[55] Hashmi S , Britton C , Liu J , Guiliano DB , Oksov Y , Lustigman S . Cathepsin L is essential for embryogenesis and development of *Caenorhabditis elegans* . J Biol Chem. 2002;277(5):3477‐3486. 11707440 10.1074/jbc.M106117200

[56] Hemmings KE , Maruthini D , Vyjayanthi S , et al. Amino acid turnover by human oocytes is influenced by gamete developmental competence, patient characteristics and gonadotrophin treatment. Hum Reprod. 2013;28(4):1031‐1044. 23335609 10.1093/humrep/des458 PMC3600837

[57] Leese HJ , McKeegan PJ , Sturmey RG . Amino acids and the early mammalian embryo: origin, fate, function and life‐long legacy. Int J Environ Res Public Health. 2021;18(18):9874. 10.3390/ijerph18189874 PMC8467587 34574797

[58] Van Winkle LJ . Amino acid transport and metabolism regulate early embryo development: species differences, clinical significance, and evolutionary implications. Cells. 2021;10(11):3154. 10.3390/cells10113154 PMC8618253 34831375

[59] Imschenetzky M , Puchi M , Morin V , Medina R , Montecino M . Chromatin remodeling during sea urchin early development: molecular determinants for pronuclei formation and transcriptional activation. Gene. 2003;322:33‐46. 14644495 10.1016/j.gene.2003.08.024

[60] Morin V , Sanchez‐Rubio A , Aze A , et al. The protease degrading sperm histones post‐fertilization in sea urchin eggs is a nuclear cathepsin L that is further required for embryo development. PLoS One. 2012;7(11):e46850. 23144790 10.1371/journal.pone.0046850 PMC3489855

[61] Puchi M , Quinones K , Concha C , et al. Microinjection of an antibody against the cysteine‐protease involved in male chromatin remodeling blocks the development of sea urchin embryos at the initial cell cycle. J Cell Biochem. 2006;98(2):335‐342. 16408295 10.1002/jcb.20800

[62] Concha C , Monardes A , Even Y , et al. Inhibition of cysteine protease activity disturbs DNA replication and prevents mitosis in the early mitotic cell cycles of sea urchin embryos. J Cell Physiol. 2005;204(2):693‐703. 15795898 10.1002/jcp.20338

[63] Kaasik A , Rikk T , Piirsoo A , Zharkovsky T , Zharkovsky A . Up‐regulation of lysosomal cathepsin L and autophagy during neuronal death induced by reduced serum and potassium. Eur J Neurosci. 2005;22(5):1023‐1031. 16176344 10.1111/j.1460-9568.2005.04279.x

[64] Pucer A , Castino R , Mirkovic B , et al. Differential role of cathepsins B and L in autophagy‐associated cell death induced by arsenic trioxide in U87 human glioblastoma cells. Biol Chem. 2010;391(5):519‐531. 20302512 10.1515/BC.2010.050

[65] Sun M , Ouzounian M , de Couto G , et al. Cathepsin‐L ameliorates cardiac hypertrophy through activation of the autophagy‐lysosomal dependent protein processing pathways. J Am Heart Assoc. 2013;2(2):e000191. 23608608 10.1161/JAHA.113.000191 PMC3647266

[66] Wei DH , Jia XY , Liu YH , et al. Cathepsin L stimulates autophagy and inhibits apoptosis of ox‐LDL‐induced endothelial cells: potential role in atherosclerosis. Int J Mol Med. 2013;31(2):400‐406. 23229094 10.3892/ijmm.2012.1201

[67] Elahi F , Lee H , Lee J , et al. Effect of rapamycin treatment during post‐activation and/or in vitro culture on embryonic development after parthenogenesis and in vitro fertilization in pigs. Reprod Domest Anim. 2017;52(5):741‐748. 28397300 10.1111/rda.12974

[68] Lee J , Cheng X , Swails JM , et al. CHARMM‐GUI input generator for NAMD, GROMACS, AMBER, OpenMM, and CHARMM/OpenMM simulations using the CHARMM36 additive force field. J Chem Theory Comput. 2016;12(1):405‐413. 26631602 10.1021/acs.jctc.5b00935 PMC4712441

[69] Moura MT , Latorraca LB , Paula‐Lopes FE . Contextualizing autophagy during gametogenesis and preimplantation embryonic development. Int J Mol Sci. 2021;22(12):6313. 10.3390/ijms22126313 PMC8231133 34204653

[70] Pan Y , Wang M , Wang L , et al. Interleukin‐1 beta induces autophagy of mouse preimplantation embryos and improves blastocyst quality. J Cell Biochem. 2020;121(2):1087‐1100. 31453635 10.1002/jcb.29345

[71] Shen XH , Jin YX , Liang S , et al. Autophagy is required for proper meiosis of porcine oocytes maturing in vitro. Sci Rep. 2018;8:8. 30135500 10.1038/s41598-018-29872-y PMC6105682

[72] Song BS , Yoon SB , Kim JS , et al. Induction of autophagy promotes preattachment development of bovine embryos by reducing endoplasmic reticulum stress. Biol Reprod. 2012;87(1):1‐11. 10.1095/biolreprod.111.097949 22539678

[73] Tsukamoto S , Kuma A , Murakami M , Kishi C , Yamamoto A , Mizushima N . Autophagy is essential for preimplantation development of mouse embryos. Science. 2008;321(5885):117‐120. 18599786 10.1126/science.1154822

[74] Hale BJ , Li Y , Adur MK , Keating AF , Baumgard LH , Ross JW . Characterization of the effects of heat stress on autophagy induction in the pig oocyte. Reprod Biol Endocrinol. 2021;19(1):107. 34243771 10.1186/s12958-021-00791-4 PMC8268447

[75] Latorraca LB , Feitosa WB , Mariano C , et al. Autophagy is a pro‐survival adaptive response to heat shock in bovine cumulus‐oocyte complexes. Sci Rep. 2020;10(1):13711. 32792582 10.1038/s41598-020-69939-3 PMC7426922

[76] Buck MR , Karustis DG , Day NA , Honn KV , Sloane BF . Degradation of extracellular‐matrix proteins by human cathepsin B from normal and tumour tissues. Biochem J. 1992;282(Pt 1):273‐278. 1540143 10.1042/bj2820273 PMC1130919

[77] Mort JS , Buttle DJ . Cathepsin B. Int J Biochem Cell Biol. 1997;29(5):715‐720. 9251238 10.1016/s1357-2725(96)00152-5

[78] Pezhman M , Hosseini SM , Ostadhosseini S , Rouhollahi Varnosfaderani S , Sefid F , Nasr‐Esfahani MH . Cathepsin B inhibitor improves developmental competency and cryo‐tolerance of in vitro ovine embryos. BMC Dev Biol. 2017;17(1):10. 28676034 10.1186/s12861-017-0152-2 PMC5496377

[79] Liu MH , Liu AJ , Qi X , et al. Excessive expression and activity of cathepsin B in sheep cumulus cells compromises oocyte developmental competence. Small Ruminant Res. 2017;151:82‐89.

[80] Liang S , Jiang H , Shen XH , Zhang JB , Kim NH . Inhibition of cathepsin B activity prevents deterioration in the quality of in vitro aged porcine oocytes. Theriogenology. 2018;116:103‐111. 29800805 10.1016/j.theriogenology.2018.04.035

[81] Kirschke H , Cathepsin L . Handbook of Proteolytic Enzymes. Vol 1 and 2. 3rd ed.; Academic Press; 2013:1808‐1817.

[82] Joyce JA , Baruch A , Chehade K , et al. Cathepsin cysteine proteases are effectors of invasive growth and angiogenesis during multistage tumorigenesis. Cancer Cell. 2004;5(5):443‐453. 15144952 10.1016/s1535-6108(04)00111-4

[83] Raes A, Wydooghe E, Pavani KC , et al. Cathepsin‐L secreted by high‐quality bovine embryos exerts an embryotrophic effect In vitro. Int J Mol Sci. 2023;24(7):6563. 10.3390/ijms24076563 PMC10094850 37047535

[84] Kidder GM , Vanderhyden BC . Bidirectional communication between oocytes and follicle cells: ensuring oocyte developmental competence. Can J Physiol Pharmacol. 2010;88(4):399‐413. 20555408 10.1139/y10-009 PMC3025001

[85] Brower PT , Schultz RM . Intercellular communication between granulosa cells and mouse oocytes: existence and possible nutritional role during oocyte growth. Dev Biol. 1982;90(1):144‐153. 7199496 10.1016/0012-1606(82)90219-6

[86] Eppig JJ , O'Brien M , Wigglesworth K . Mammalian oocyte growth and development in vitro. Mol Reprod Dev. 1996;44(2):260‐273. 9115726 10.1002/(SICI)1098-2795(199606)44:2<260::AID-MRD17>3.0.CO;2-6

[87] Marley MSD , Givens MD , Galik PK , Riddell KP , Stringfellow DA . Lactoferrin from bovine milk inhibits bovine herpesvirus 1 in cell culture but suppresses development of in vitro‐produced bovine embryos. Anim Reprod Sci. 2009;112(3–4):423‐429. 18586420 10.1016/j.anireprosci.2008.05.075

[88] Eipper S , Steiner R , Lesner A , et al. Lactoferrin is an allosteric enhancer of the proteolytic activity of Cathepsin G. PLoS One. 2016;11(3):e0151509. 26986619 10.1371/journal.pone.0151509 PMC4795699

[89] An Q , Peng W , Cheng Y , et al. Melatonin supplementation during in vitro maturation of oocyte enhances subsequent development of bovine cloned embryos. J Cell Physiol. 2019;234(10):17370‐17381. 30786018 10.1002/jcp.28357

[90] Graf A , Krebs S , Zakhartchenko V , Schwalb B , Blum H , Wolf E . Fine mapping of genome activation in bovine embryos by RNA sequencing. Proc Natl Acad Sci U S A. 2014;111(11):4139‐4144. 24591639 10.1073/pnas.1321569111 PMC3964062

[91] Mamo S , Mehta JP , McGettigan P , et al. RNA sequencing reveals novel gene clusters in bovine conceptuses associated with maternal recognition of pregnancy and implantation. Biol Reprod. 2011;85(6):1143‐1151. 21795669 10.1095/biolreprod.111.092643

[92] Reyes JM , Chitwood JL , Ross PJ . RNA‐Seq profiling of single bovine oocyte transcript abundance and its modulation by cytoplasmic polyadenylation. Mol Reprod Dev. 2015;82(2):103‐114. 25560149 10.1002/mrd.22445 PMC4651626

[93] Teng CT . Lactoferrin gene expression and regulation: an overview. Biochem Cell Biol. 2002;80(1):7‐16. 11908645 10.1139/o01-215

[94] Massa E , Pelusa F , Lo Celso A , et al. Lactoferrin levels in cervical fluid fromin vitro fertilization (IVF) patients—correlation with IVF parameters. Biochem Cell Biol. 2021;99(1):91‐96. 32476453 10.1139/bcb-2020-0098

[95] Cimadomo D , Fabozzi G , Vaiarelli A , Ubaldi N , Ubaldi FM , Rienzi L . Impact of maternal age on oocyte and embryo competence. Front Endocrinol (Lausanne). 2018;9:327. 30008696 10.3389/fendo.2018.00327 PMC6033961

