# Supplementary figures and images for: Cathepsin L regulates oocyte meiosis and preimplantation embryo development
Source: Cell Prolif. 2023 Jul 7;57(1):e13526. doi: 10.1111/cpr.13526 (PMC10771118; doi:10.1111/cpr.13526)

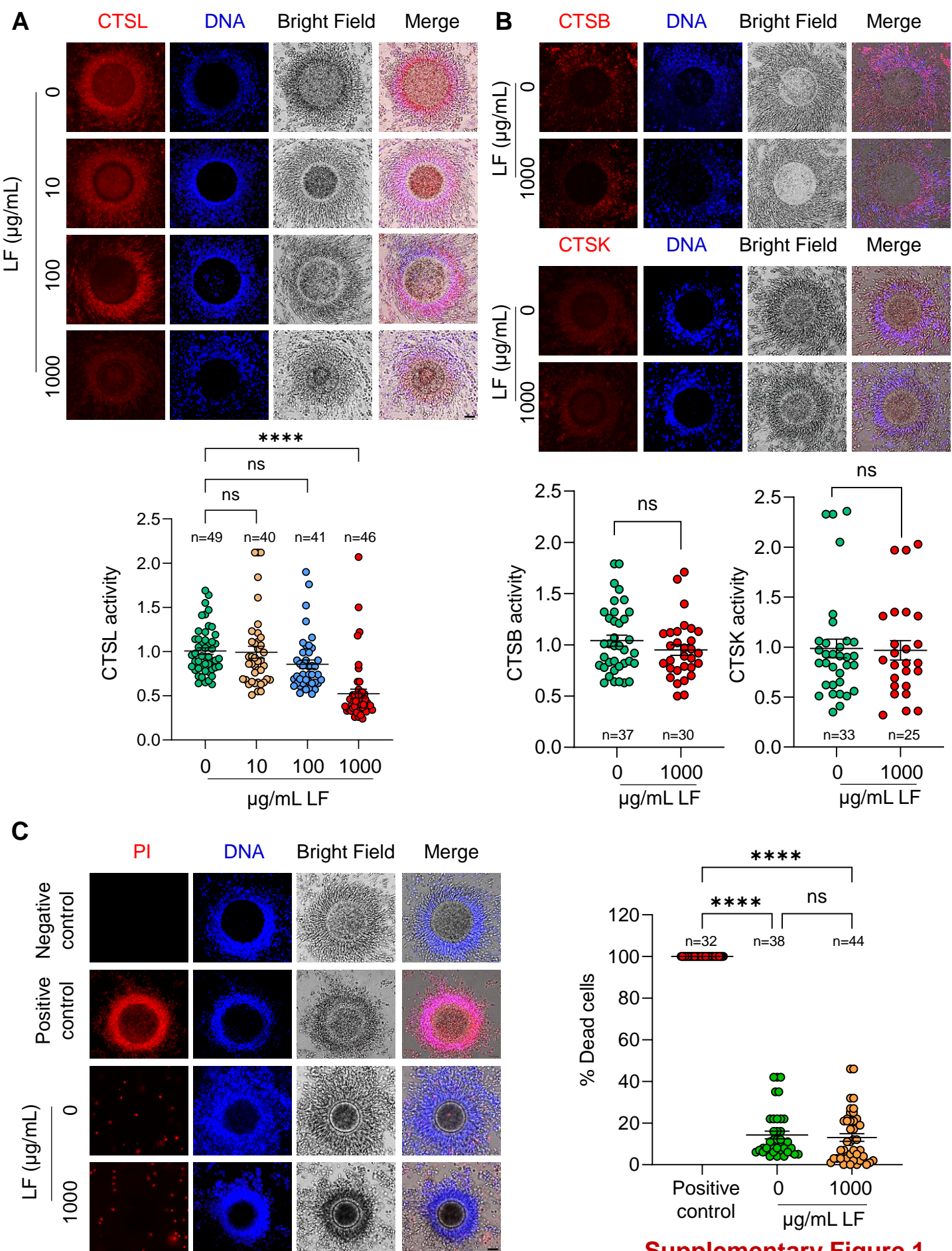

Supplementary Figure 1

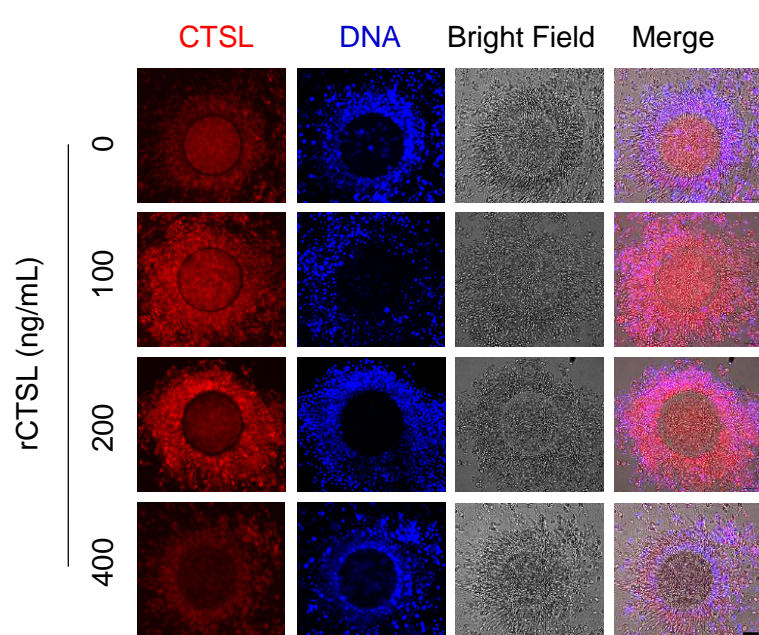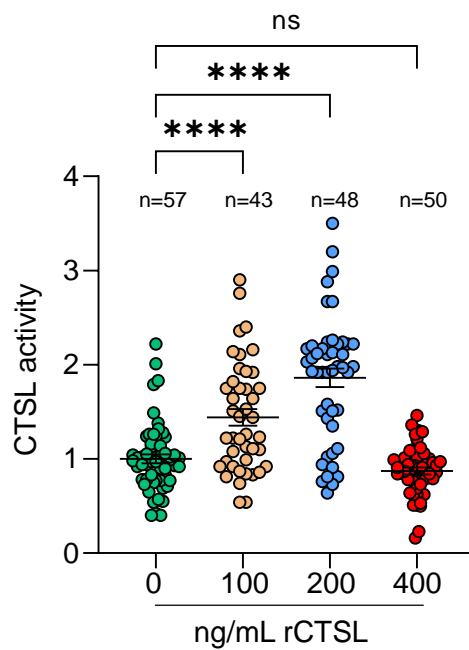

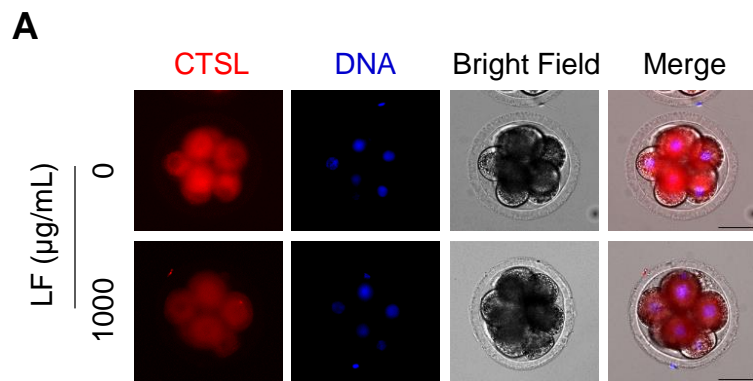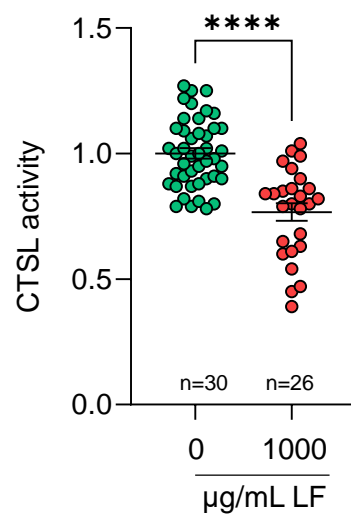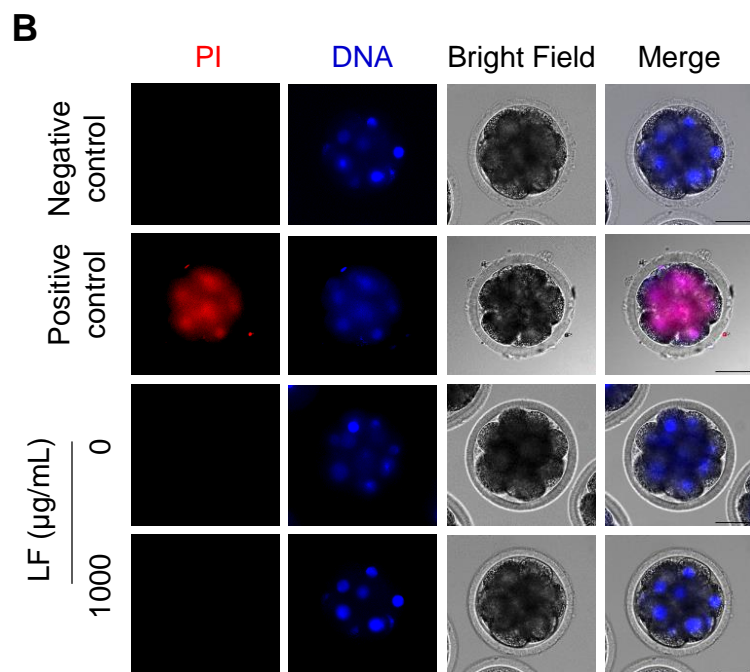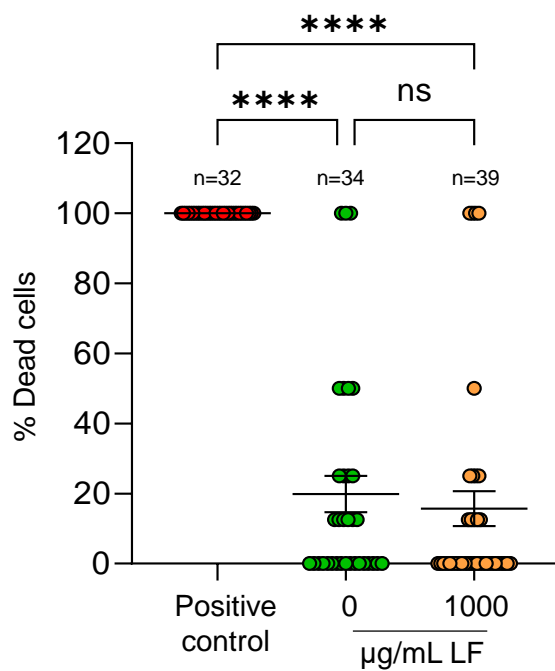

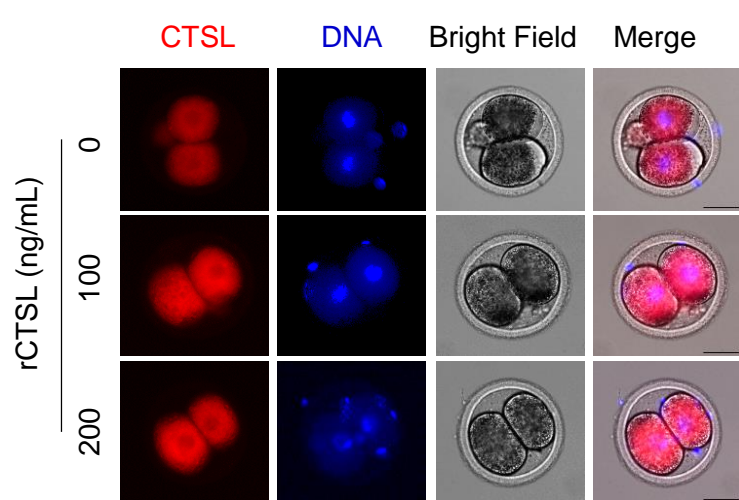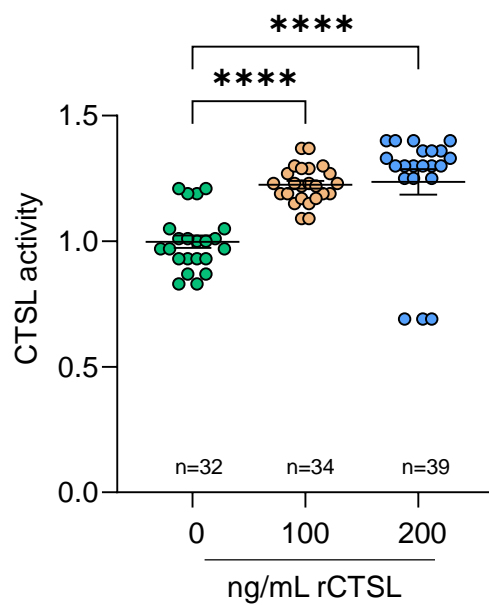

Supplement: Supplementary file 1 — FIGURE S1: Lactoferrin inhibits CTSL activity in COCs without disturbing cell livability. Bovine COCs were in vitro matured with LF (at 0, 10, 100 or 1000 μg/mL) for 22–24 h followed by detecting intracellular CTSL activity (A), CTSB/K activity (B) or the assessing the cell livability (B) in LF‐treated COCs. Positive‐control COCs were treated with DNase (50 U/mL) for 1 h prior to PI/Hoechst 33342 staining, while negative‐control COCs were stained with Hoechst 33342 (PI staining was excluded), Scale bar represents 50 μm. The data of three independent experiments are presented as mean ± SEM. The total number of analysed COCs was specified in each graph. Asterisks denote a significant variance (**** means p < 0.0001) between the different groups when compared using one‐way ANOVA followed by Tukey's multiple comparisons test. The number of replicates was included in our statistical model; there was no significant variance between the different replicates. FIGURE S2: Recombinant human CTSL enhances CTSL activity in COCs. Bovine COCs were in vitro matured with rCTSL (at 0, 100, 200 or 400 ng/mL) for 22–24 h followed by the detection of intracellular CTSL activity. Scale bar represents 50 μm. The data of three independent experiments are presented as mean ± SEM. The total number of analysed COCs was specified in each graph. Asterisks denote a significant variance (** means p < 0.01, and **** means p < 0.0001) between the different groups when compared using one‐way ANOVA followed by Tukey's multiple comparisons test. The number of replicates was included in our statistical model; there was no significant variance between the different replicates. FIGURE S3: Lactoferrin inhibits CTSL activity in early embryos without disturbing cell livability. Cleaved embryos at 60 hpi were in vitro cultured with LF (at 0 or 1000 μg/mL) for 24 h, followed by the detection of intracellular CTSL activity (A) or the assessment of cell livability (B) in LF‐treated embryos. Positive‐control emb [file CPR-57-e13526-s001.pdf]
